# Comparing the Effect of Biochar and Poultry Manure on the Metabolomic Profile of Tomatoes (*Solanum lycopersicum* L.)

**DOI:** 10.3390/metabo16070504

**Published:** 2026-07-17

**Authors:** Rolivhuwa Carol Mudau, Lufuno Ethel Nemadodzi

**Affiliations:** Department of Agriculture and Animal Health, University of South Africa, Private Bag X6, Florida 1710, South Africa; 69104670@mylife.unisa.ac.za

**Keywords:** 1H-NMR, biochar, poultry manure, metabolites, allantoin, cellobiose

## Abstract

**Background**: In the coming decades, the agricultural system will increasingly rely on organic amendments to sustain crop production, maintain soil health and ensure long-term food security. While inorganic fertilizers remain widely used for vegetable production due to their rapid nutrient availability, their prolonged application has been shown to degrade soil quality, disrupt microbial communities and limit the nutritional quality of harvested produce. Consequently, there is a growing need to explore organic alternatives such as biochar and poultry manure that can enhance both crop productivity and functional quality. **Objective**: This study aimed to determine the influence of different application rates of biochar (5/T_1_, 10/T_2_, and 20/T_3_ t/ha), poultry manure (10/T_1_, 20/T_2_, 30/T_3_ t/ha), and NPK (2:3:4) as control on the metabolomic profile of tomato fruit. **Methods**: The metabolomic profile was determined using 1H-nuclear magnetic resonance (NMR). **Results**: Common metabolites across biochar rates included allantoin, asparagine and betaine, while rate-specific released metabolites such as inosine (T_1_), epicatechin (T_2_) and kynurenine (T_3_) were also identified. Similarly, poultry manure showed an array of metabolites at the highest application rate, with metabolites such as cellobiose, creatinine and maltose, while histamine (T_1_) and ADP (T_3_) were also detected as distinct metabolites.

## 1. Introduction

Tomato (*Solanum lycopersicum* L.) belongs to the Solanaceae family [1] and is distinguished from other vegetables due to its high nutritional profile and widespread consumption [2]. It is grown worldwide [3], can be cultivated in protected cultures and open fields [4], and may thrive in a variety of climates, from hot, humid tropical to moderate [5]. Different tomato cultivars such as Roma tomatoes, cherry tomatoes, heirloom tomatoes, and beefsteak are commonly grown around the world including in Africa [6]. However, in South Africa, the Roma VF cultivar, also known as Jam tomato, is preferred by most farmers for its resistance to disease, which can reduce the need for chemical treatments and ultimately help improve yield [7].

Fresh tomato fruits have been reported to lower the risks of cardiovascular illnesses and some types of cancer, such as prostate, lung, and stomach [8]. A study by Giovannucci [9] found that men consuming ≥10 servings/week of tomatoes had a 35% lower chance of prostate cancer risk.

A study by Li et al. [10] reported that biochar addition improved soil properties and increased tomato yield, with the highest yield obtained at an application rate of 40 t ha^−1^. Ref. [11] demonstrated that biochar application increased tomato fruit yield and improved fruit quality, increasing total acidity, Vitamin C, and lycopene content. Furthermore, a recent study found that biochar application enhanced nutrient uptake, photosynthetic traits, and tomato yield, even under reduced nitrogen application [12].

Poultry manure is a mixture of bedding materials (wood shavings, sawdust, and peanut shells), bird excreta, feather barbules, feed residues, and chemical treatments (alum, sodium bisulphate) cleaned out of chicken-rearing facilities [13]. Jones [14] stated that poultry manure is usually richer in nitrogen than other livestock manure because birds have a common duct for the elimination of urine and feces. It is also very cheap and effective as a source of N for sustainable crop production [15].

Plants produce a vast and diverse array of organic compounds, the majority of which do not appear to participate directly in growth and development [16]. Nemadodzi et al. [17] defined metabolites as the end products of a variety of cellular processes which provide high-throughput characterization and quantification of living organisms, and as a result, are increasingly applied to the areas of system biology, drug discovery, pharmaceutical research, early disease detection, toxicology, and food science. According to Nemadodzi and Managa [18], metabolomics has been widely applied in several human and plant studies, and is slowly gaining attention in soil science studies. However, due to few studies conducted using advanced metabolomics tools on African leafy vegetables such as *amaranth*, spider plant, jute mallow, and pumpkin leaves, there is little and/ or limited scientific literature recorded on their biological constituents [19]. According to Tiwari [16], primary and secondary metabolites are the intermediates of small molecules, and they have several functions such as catalytic activity, stimulatory and inhibitory influence on enzymes, structure, signaling, energy and defense mechanisms. Different metabolomics tools such as nuclear magnetic resonance (NMR) spectroscopy and liquid chromatography–mass spectrometry (LC-MS) analyses are commonly used to determine the metabolite profile of various samples [19]. The advantages of using NMR include its non-destructiveness, unbiasedness, quantitative, and uncomplicated sample preparation, and it may be used to study compounds that are difficult to detect using gas chromatography–mass spectrometry (GC-MS) or LC-MS [17,19].

Primary metabolites perform their function as signaling molecules to trigger defense response by signal transduction and pathogen recognition processes [20]. Production of primary metabolites occurs during the active growth phase (trophophase) and begins in the presence of required nutrients in a growth medium. Primary metabolism is important for the growth, development and reproduction of cells, and the regulation of carbohydrates, protein and lipids in response to infection by pathogens [21]. Plant growth and yield are affected by primary and secondary metabolism during pathogen infection [22]. Energy supply is compulsory for the defense response [23]. Pathogens easily manipulate plants’ metabolism in a limited supply of nutrients, which increases the nutrient demand for assimilation [24]. The role of primary metabolic pathways in the regulation of plant defense responses for development and growth is very little known [25].

Secondary metabolites are organic compounds that are not directly involved in the normal growth, development, or reproduction of an organism [26]. They are often produced in a specific stage of growth, a dormant phase (survival mode), and by limited taxonomic groups, with unusual structures, and are usually members of related families [27]. Secondary metabolites also play an important role in plant defense against herbivory [28] and other interspecific defenses [29]. According to a study by Yang et al. [30], over 2100 metabolites with 125 modification types have been annotated in tomatoes, highlighting their biochemical complexity. Another study by Sun et al. [31] detected 847 metabolites in tomato fruit under salt stress, including primary metabolites such as lipids, amino acids, sugars, and organic acids, and secondary metabolites such as phenolic acids, alkaloids, and flavonoids. Sing et al. [32] identified apigenin-7-glucoside, uridine, homocyctine, adenosyl-homocysteine, guanosine cyclic monophosphate (cGMP), tyrosine, pantothenic acid, adenosine, adenosine diphosphate, azmaline and riboflavin. Currently, there is a huge gap in metabolomic studies on tomato varieties, the impact of growth conditions, fertilizer (organic and inorganic) application, and irrigation systems in South Africa. The initial hypothesis of the study was that the tomato plant cultivated with different rates of biochar and poultry manure would not affect the metabolomic profile of the tomato fruit. Therefore, the current study aimed to determine the influence of biochar and poultry manure on the metabolomic profile of open-field grown tomatoes.

## 2. Materials and Methods

The open-field study was conducted at Ndima Cash Crop Farms, Westonaria, South Africa (26.2505° S, 27.7166° E). The region experiences average minimum temperatures of 3 and maximum temperatures of 7 °C (June/July) and 15 (lowest) to 28 °C (highest) (January). The experiment used a randomized complete block design (RCBD) with three treatments replicated 9 times in 3 × 3 m plots. Treatments consisted of pinewood biochar (produced under high temperature >5500 °C) applied at 5, 10, and 20 t/ha^−1^ as prescribed by Musumuvhi [33], and poultry manure applied at 10, 20, and 30 t/ha^−1^ as prescribed by Agaba et al. [34]. The biochemical analysis of pinewood biochar manure and poultry manure are indicated in Table 1 and Table 2 respectively. Amendments were incorporated into the soil (6 cm depth) one week before planting. Roma VF tomato seedlings were transplanted at 0.5 m spacing. The experiment was rain-fed, but survival irrigation was applied when necessary, using sprinklers, and lasted three months (December 2024–April 2025).

### 2.1. Fruit Collection and Preparations

The red ripe fruits were manually collected at 12 weeks harvest, grated and taken to the Council for Scientific and Industrial Research (CSIR) for metabolomics analysis.

### 2.2. NMR Metabolomic Analysis

Fresh, red, ripe tomato fruit samples were subjected to the NMR metabolomic analysis protocol, following Kim et al. [35]. Deuterated methanol (CD_3_OD), non-deuterated KH2PO4 sodium deuterium oxide (NaOD), trimethylsilyl propionic acid sodium salt (TSP) and deuterium oxide (D_2_O) were supplied by Sigma-Aldrich (Darmstadt, Germany). The buffer was prepared by adding 1.232 g KH_2_PO_4_ to 100 mL of D_2_O with 10 mg TSP (0.01%) added as a reference standard. The pH of the solution was adjusted to 6. A 50 mg fresh fruit sample (unpeeled and grated) was transferred into each of the 2 mL Eppendorf tubes. The samples were extracted with 750 µL CD_3_OD and 750 µL KH_2_PO4 buffer in D_2_O (pH 6.0) containing 0.01% TSP. The Eppendorf tubes were then vortexed for 1 min at room temperature and then ultrasonicated for 20 min without heating. Thereafter, the solution was centrifuged for another 15 min at 10,000 revolutions per minute (rpm) to separate the supernatant from the precipitate. The supernatant was then transferred to standard 5 mm NMR tubes and subjected to 1H-NMR analysis. The 1H-NMR measurements were performed on a Varian 600 MHz spectrometer with a frequency of 599.74 MHz. The acquisition time of each 1H-NMR spectrum was 7 min, with 32 scans and a width of 20 ppm. The technical rates were replicated 5 times. All spectra were phase-corrected, and data normalization was done at the largest peak. Gradient shimming was used to improve magnetic field homogeneity and binned at 0.04 ppm using MestReNova [36]. The NMR spectrum width of 0–10 ppm and pre-processed data were statistically analyzed with SIMCA 18.0.2 (Umetrics, Umea, Sweden).

### 2.3. Statistical Analysis

Two (2) statistical models were used: unsupervised principal component analysis (PCA-X) and supervised orthogonal partial least square discriminant analysis (OPLS-DA) [37,38]. The water and methanol peaks were excluded from the variables between 3.28–3.34 and 4.6–5 respectively, and scaling was done using the Pareto method.

### 2.4. Annotation and Detection of Metabolites

Furthermore, the raw NMR data were pre-processed with Mestronova version 15.0.1 to detect the NMR peaks in parts per million (ppm). Metabolite annotation and identification were carried out using Chenomx NMR suite 11.0 (Edmonton, AB, Canada), which has specific characteristics (also known as values) for a variety of metabolites [19]. The Chenomx Library manager was used to obtain all compounds, and Chenomx compound builder version 11 was used to create 1D NMR compounds by group of peaks shape and/or signal region. External database references, such as Human Metabolome Data Base (HMDB), were also used to compare and confirm the detected and quantified metabolites [17].

## 3. Results

### 3.1. The Impact of Biochar Rates on the Metabolomic Profile of Tomato

**Figure 1:**
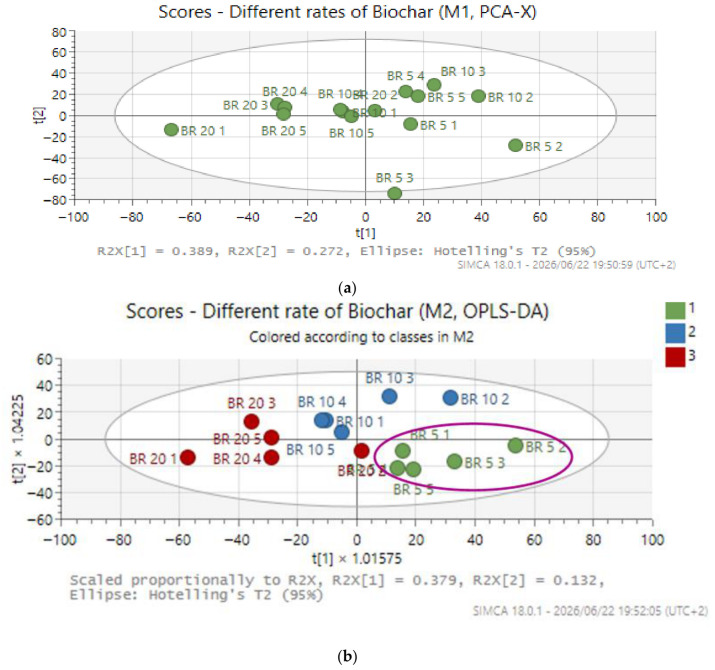
(**a**). PCA-X model scatter plot showing clusters of fresh tomato samples treated with 5 (T_1_), 10 (T_2_), and 20 (T_3_) t/ha of biochar, herein referred to as BR5, BR10, BR20, respectively. (**b**). OPLS-DA model showing a clear separation of the biochar rate (5tons/ha), herein referred to as BR 5 (T_1_), from the two treatments.

NMR data processing was carried out using SIMCA 18.0.2, and the results indicated that the OPLS-DA (supervised) model showed a distinct separation between the treatments, as shown in Figure 1b.

The R^2^ and Q^2^ values as well as their components derived from OPLS-DA are indicated in Table 3.

Distinct metabolites such as 3-hydroxylsova-lerate, inosine, choline, glycylproline, and nicotinic acid adenine dinucleotide per T_1_ biochar rate were annotated and compared to external databases such as HMDB, as explained in the Section 2 (see Table 4).

**Figure 2:**
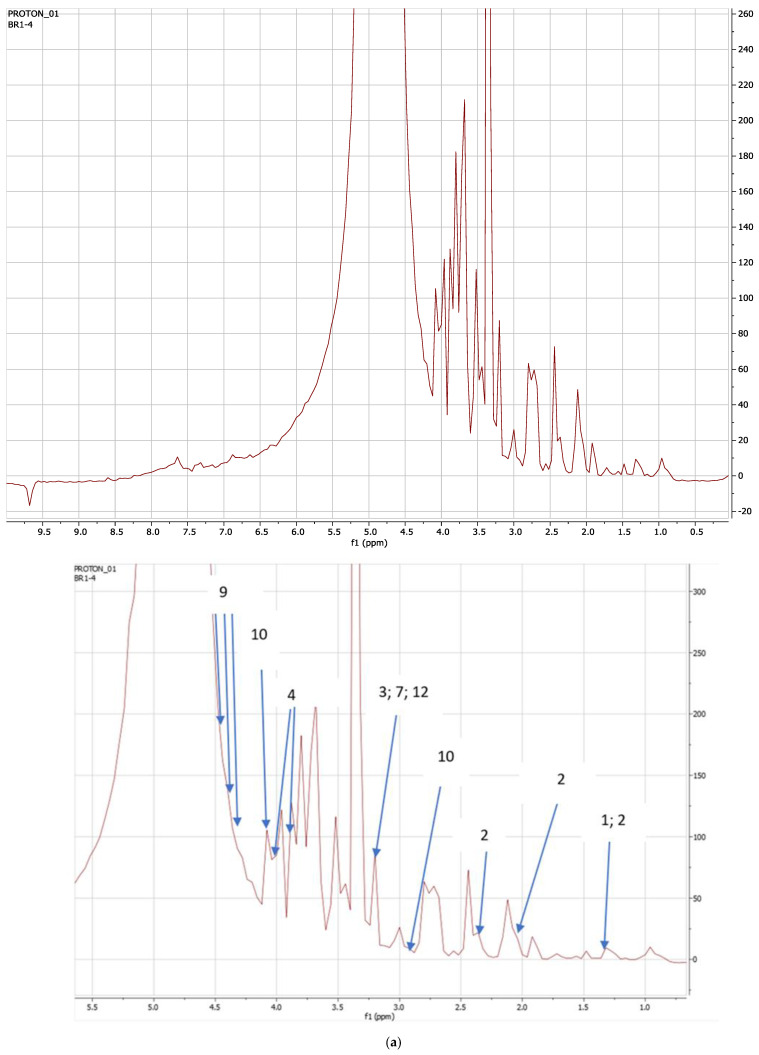

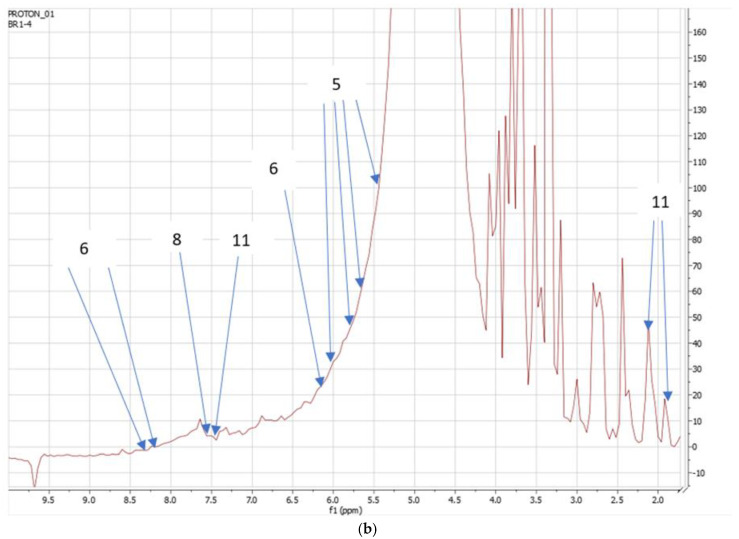
The full NMR spectra from biochar applied at a rate of 5 (T_1_) tons/ha. (**a**) The NMR spectra showing distinct peaks detected on fresh tomato when biochar was applied at 5 t/ha (T_1_): 1. 3-hydroxy-3 methylglutarate (1.3 ppm); 2. 3-hydroxylsovalerate (2.4, 2.3, 1.3 ppm); 3. Choline (3.2 ppm); 4. Glycylproline (4.0, 3.9 ppm); 7. Malonate (3.2 ppm); 9. Nicotinic acid adenine dinucleotide (4.5, 4.4, 4.3 ppm); 10. N-methylhydantoin (4.1, 2.9 ppm); 12. O-acetylcholine (3.2, 2.1 ppm). (**b**) The NMR spectra showing distinct peaks detected on fresh tomato when biochar was applied at 5 t/ha: 5. Homocitrulline (6.0, 5.8, 5.7, 5.4 ppm); 6. Inosine (8.3, 8.2, 6.1 ppm); 8. Nicotinate (7.6 ppm); 11. Na-acetyllysine (7.5, 2.1, 2.0 ppm).

To detect and identify the secondary metabolites of field-grown tomatoes, NMR spectra for the current study were developed using Mestrenova software, showing different peaks in the NMR ppm regions, as indicated in Figure 2a,b.

Distinct metabolites such as Acetamide, AMP, and Anserine were also detected per T_2_ biochar rate and compared to HMDB, as explained in Materials and Methods (see Table 5).

**Figure 3:**
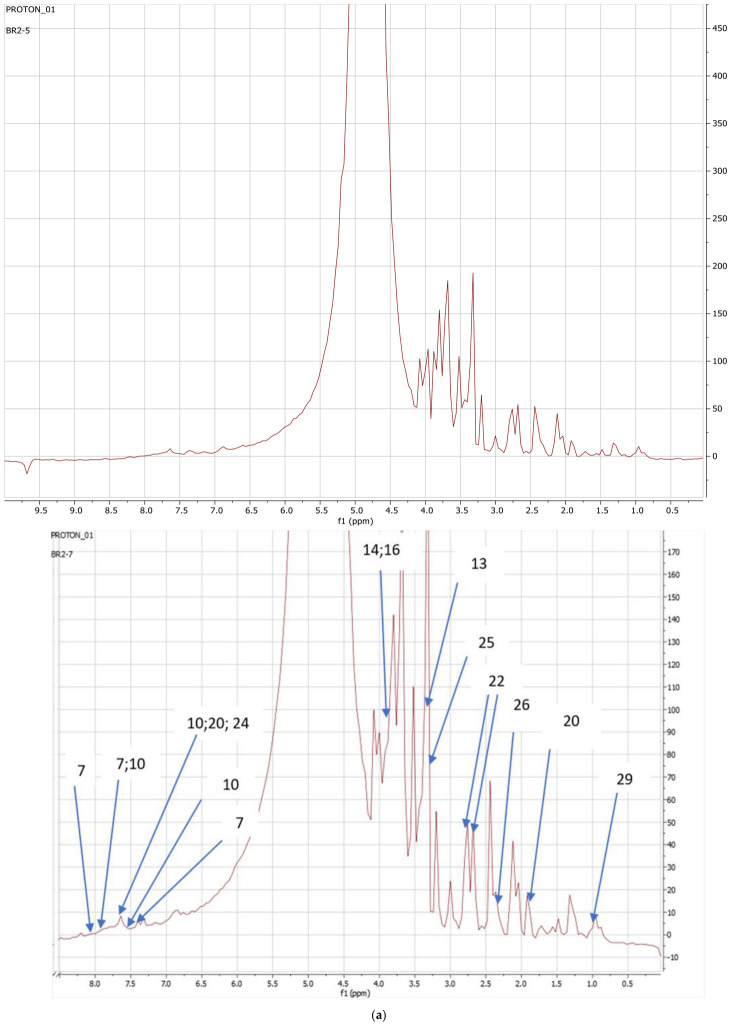

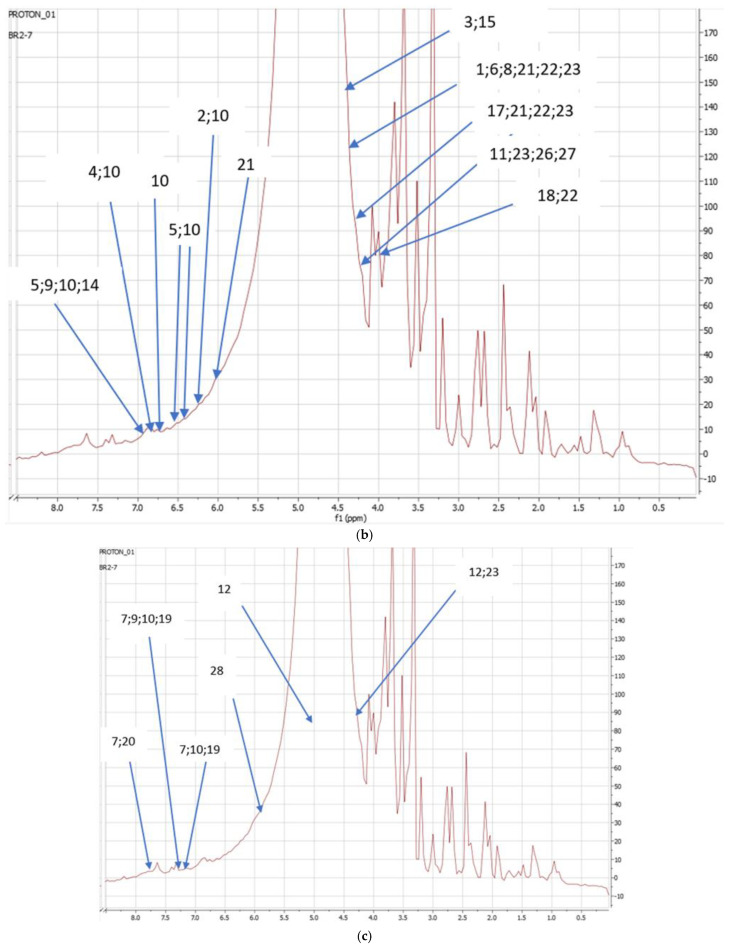
Full NMR spectrum obtained from a 10 tons/ha (T_2_) of biochar application rate. (**a**) The NMR spectra showing distinct peaks detected on fresh tomato when T_2_ biochar rate was applied: 7. Anserine (7.9, 7.8, 7.7, 7.6, 7.5, 7.4, 7.3, 7.2, 7.1, 7.0, 3.8 ppm); 10. Citrulline (7.8, 7.6, 7.5, 7.3, 7.2, 7.1, 7.0, 6.9, 6.8, 6.7, 6.6, 6.5, 6.4, 6.3, 6.2, 6.1 ppm); 13. Ethylene glycol (3.7 ppm); 14. Ferulate (6.9, 6.4, 3.9 ppm); 16. Glycolate (3.9 ppm); 20. N-acetyltyrosine (7.7, 7.6, 1.9 ppm); 22. NADH (7.0, 6.9, 6.1, 4.5, 4.4, 4.3, 4.2, 4.1, 2.8, 2.7 ppm); 24. Nicotinamide-N-oxide (7.6 ppm); 25. N-phenylacetylphenylalanine (4.5, 3.6 ppm); 26. P-cresol (2.3 ppm); 29. Valine (1.0 ppm). (**b**) The NMR spectra showing distinct peaks detected on fresh tomato when T_2_ biochar rate was applied: 1. 1 methylnicotinamide (4.5 ppm); 2. 2′-deoxyguanosine (6.3 ppm); 3. 2-deoxyinosine (4.6 ppm); 4. 3-hydroxyphenylacetate (6.8 ppm); 5. Acetamide (7.7, 6.9, 6.5, 6.4 ppm); 6. AMP (4.5 ppm); 8. Carnosine (4.5 ppm); 9. Chlorogenate (7.2, 6.9, 6.4 ppm); 10. Citrulline (7.8, 7.6, 7.5, 7.3, 7.2, 7.1, 7.0, 6.9, 6.8, 6.7, 6.6, 6.5, 6.4, 6.3, 6.2, 6.1 ppm); 11. Cytidine (4.2 ppm); 14. Ferulate (6.9, 6.4, 3.9 ppm); 15. Galactose (5.3, 4.6 ppm); 17. N-acetylcysteine (4.4 ppm); 18. N-acetylglutamate (4.1 ppm); 21. NAD+ (6.1, 6.0, 4.5, 4.4 ppm); 22. NADH (7.0, 6.9, 6.1, 4.5, 4.4, 4.3, 4.2, 4.1, 2.8, 2.7 ppm); 23. NADP+ (4.5, 4.4, 4.3, 4.2 ppm); 26. P-cresol (2.3 ppm); 27. Pyroglutamate (4.2 ppm). (**c**) The NMR spectra showing distinct peaks detected on fresh tomato when T_2_ biochar rate was applied: 7. Anserine (7.9, 7.8, 7.7, 7.6, 7.5, 7.4, 7.3, 7.2, 7.1, 7.0, 3.8 ppm); 9. Chlorogenate (7.2, 6.9, 6.4); 10. citrulline (7.8, 7.6, 7.5, 7.3, 7.2, 7.1, 7.0, 6.9, 6.8, 6.7, 6.6, 6.5, 6.4,6.3, 6.2, 6.1 ppm); 12. Epicatechin (5.0, 4.3 ppm); 19. N-acetylserotonin (7.2, 7.1, 6.8 ppm); 20. N-acetyltyrosine (7.7, 7.6, 1.9 ppm); 23. NADP+ (4.5, 4.4, 4.3, 4.2 ppm); 28. UDP-galactose (6.0, 5.6, 4.3, 4.2 ppm).

To detect and identify the secondary metabolites of field-grown tomatoes, NMR spectra for the current study were developed using Mestrenova software, revealing different peaks in the NMR ppm regions, as indicated in Figure 3a–c.

Distinct metabolites such as ADP, creatinine, galactarate, and glycocholate were compared to HMDB biochar application rate of 20 t/ha (see Table 6).

**Figure 4:**
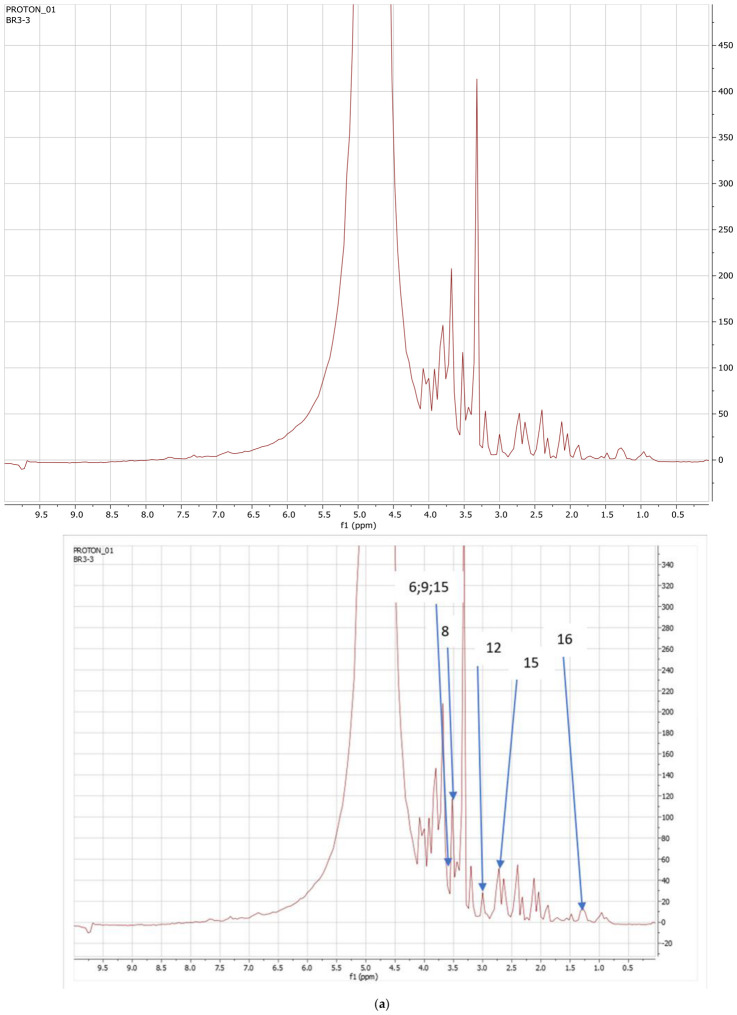

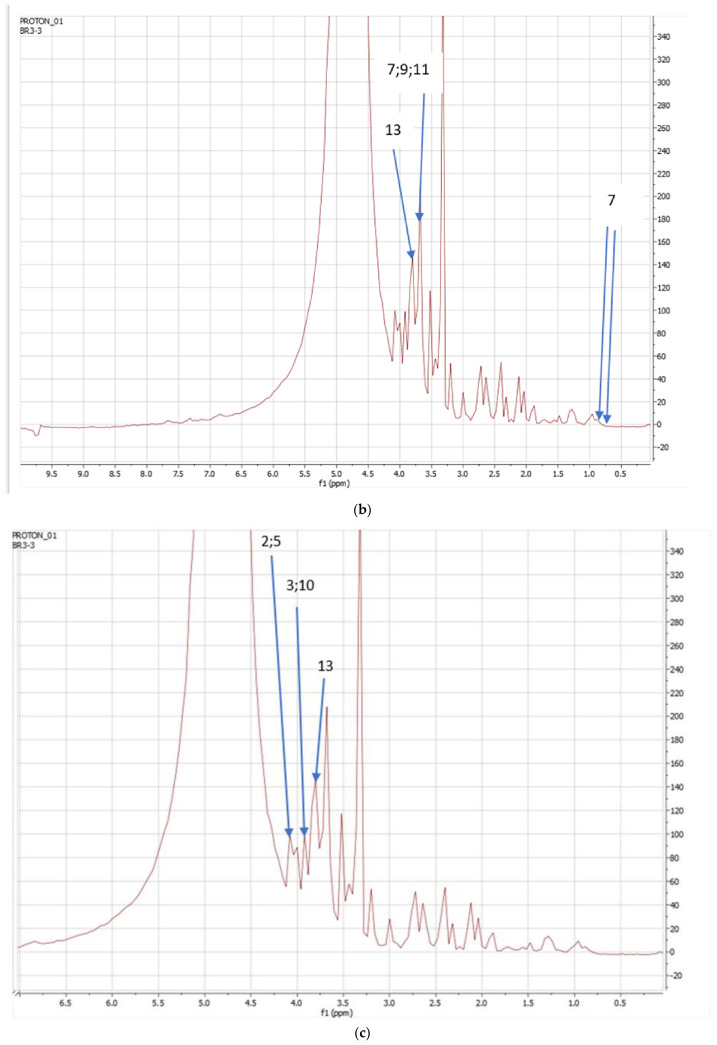

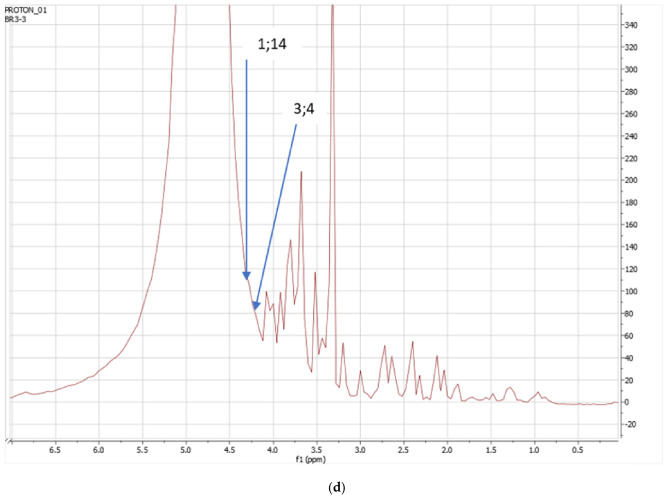
The full NMR spectra from T_3_ biochar rate. (**a**) The NMR spectra showing distinct peaks detected on fresh tomato when T_3_ biochar rate was applied: 6. Glycine (3.6 ppm); 8. Homogentisate (3.5 ppm); 9. Indole-3-acetate (3.7, 3.6 ppm); 12. N-N dimethylformamide (3.0 ppm); 15. Sarcosine (3.6, 2.7 ppm); 16. Threonine (1.3 ppm). (**b**) The NMR spectra showing distinct peaks detected on fresh tomato when T_3_ biochar rate was applied: 7. Glycocholate (3.7, 0.9, 0.7 ppm); 9. Indole-3-acetate (3.7, 3.6 ppm); 11. Kynurenine (3.7 ppm); 13. N-nitrosodimethylanine (3.0 ppm). (**c**) The NMR spectra showing distinct peaks detected on fresh tomato when T_3_ biochar rate was applied: 2. Creatinine (4.1 ppm); 3. Galactarate (4.3, 3.9); 5. Glucarate (4.1 ppm); 10. Isoeugenol (3.9 ppm); 13. N-nitrosodimethylamine (3.8 ppm). (**d**) The NMR spectra showing distinct peaks detected on fresh tomato when T_3_ biochar rate was applied: 1. ADP (4.4 ppm); 3. Galactarate (4.3, 3.9 ppm); 4. Galactonate (4.3 ppm); 14. S-adenosylhomocysteine (4.4 ppm).

To detect and identify the secondary metabolites of field-grown tomatoes, NMR spectra for the current study were developed using Mestrenova software, showing different peaks in the NMR ppm regions, as indicated in Figure 4a–d.

In addition, the results showed common metabolites released across all different rates of biochar, such as allantoin, asparagine, biotin, 1,3-dihydroxy-acetone, 1,3-dimethylurate, sucrose, arabinose, fucose, lactulose, maltose, N,N-dimethylglycine, N-acetylglutamine, cellobiose, glutamine, and ribose.

### 3.2. The Impact of Poultry Rates on the Metabolomics Profile of Tomato

**Figure 5:**
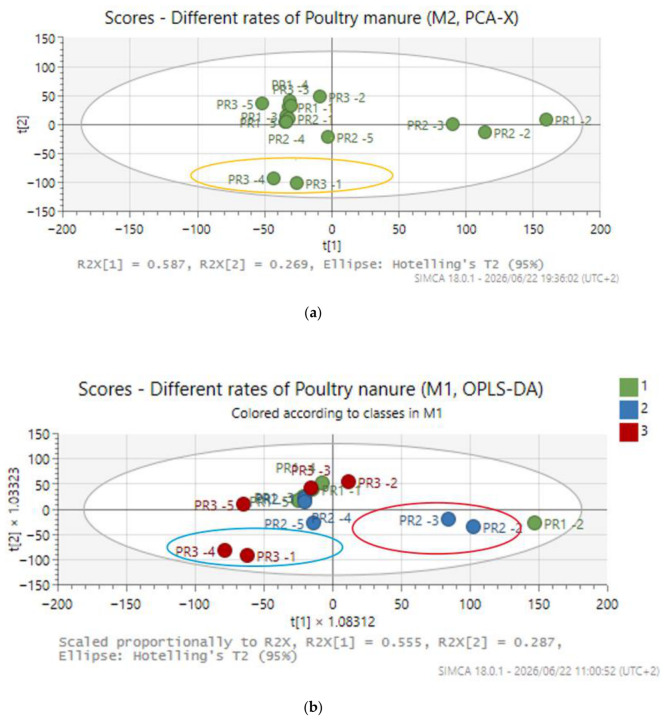
(**a**). PCA showing separation of 30 t/ha, herein referred to as PR3/T_3_, from the other treatments, herein referred to as PR1/T_1_ (10 t/ha) and PR2/T_2_ (20 t/ha). (**b**) T_2_ and T_3_ separated from the other treatments.

The same procedure explained under Section 2.2 was followed for treatment 10 (T_1_), 20 (T_2_) and 30 (T_3_) t/ha^−1^ of poultry manure to determine the different metabolites released by tomatoes, as shown in Figure 5a,b. PCA showed a separation on PR3 (T_3_), with other treatments clustered as shown in Figure 5a.

On the contrary, the OPLS-DA model showed a clear separation of PR3/ T_3_ (30 t/ha) and PR2/ T_2_ (20 t/ha), as shown in Figure 5b.

The R^2^ and Q^2^ values as well as their components derived from OPLS-DA are indicated in Table 7.

Distinct metabolites such as Histamine, Malonate, and Methionine were also detected under poultry manure application rate T_1_ and compared to HMDB (see Table 8).

**Figure 6:**
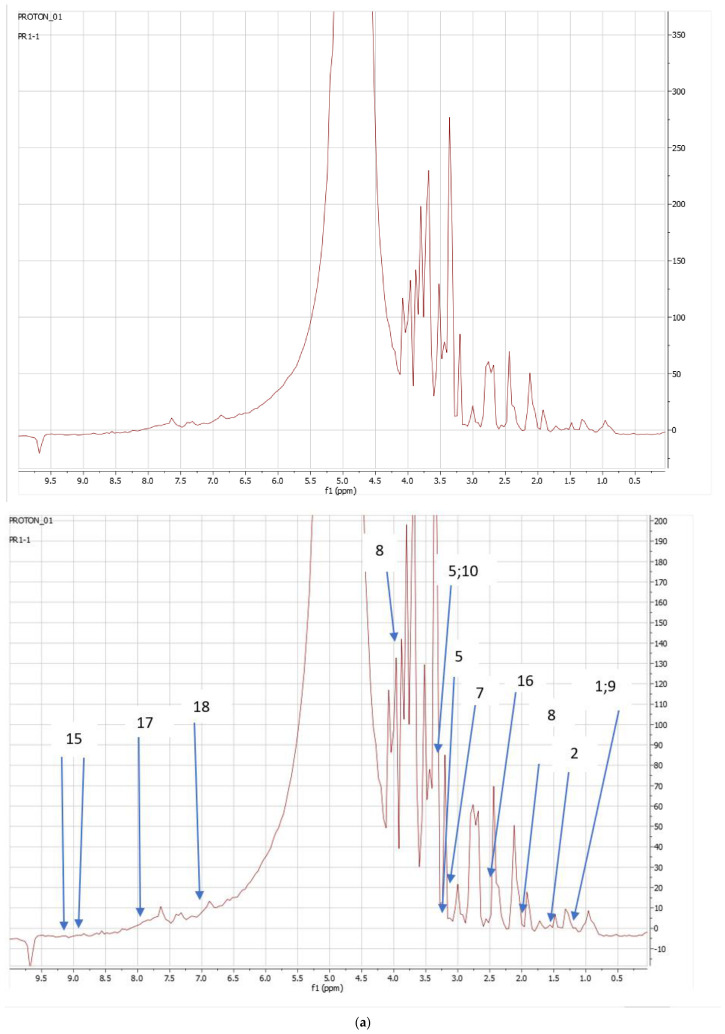

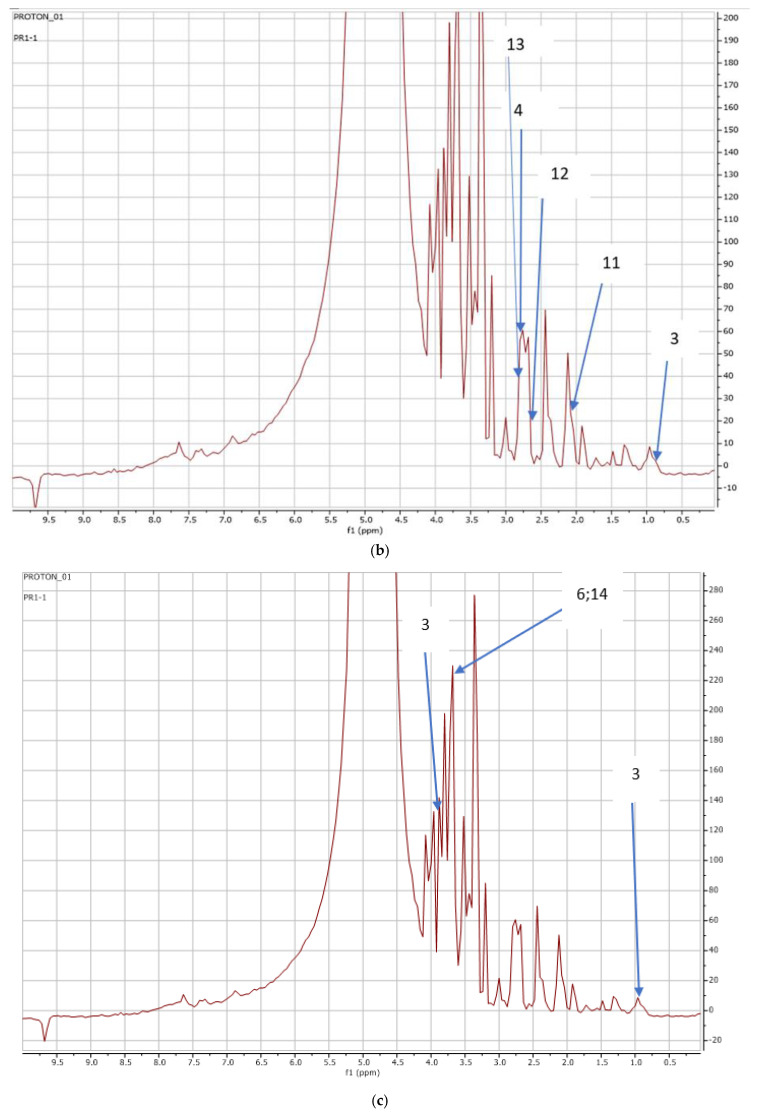
NMR full spectrum of 10 (T_1_) tons/ha of poultry manure. (**a**) The NMR spectra showing distinct peaks detected on fresh tomato when T_1_ poultry manure was applied: 1. 2-methylglutarate (1.0 ppm); 2. 2-phenylpropionate (1.4 ppm); 5. Dimethyl sulfone (3.2, 3.1 ppm); 7. Histamine (3.0 ppm); 8. Isoeugenol (3.9, 1.8 ppm); 9. Isoleucine (1.0 ppm); 10. Malonate (3.2 ppm); 15. Pyrimidine (9.1, 8.9 ppm); 16. Pyruvate (2.4 ppm); 17. Xanthine (7.9 ppm); 18. Xanthurenate (6.9 ppm). (**b**) The NMR spectra showing distinct peaks detected on fresh tomato when T_1_ poultry manure was applied: 3. 3-hydroxy-3 methylglutarate (3.8, 0.9 ppm); 4. Dimethylamine (2.7 ppm); 11. Methionine (2.1 ppm); 12. Methylamine (2.6 ppm); 13. Methylguanidine (2.8 ppm). (**c**) The NMR spectra showing distinct peaks detected on fresh tomato when T_1_ poultry manure was applied: 3. 3-hydroxy-3 methylglutarate (3.8, 0.9 ppm); 6. ethelynglycol (3.7 ppm); 14. N-phenlyacetylglycine (3.7 ppm).

To detect and identify the secondary metabolites of field-grown tomatoes, NMR spectra for the current study were developed using Mestrenova software, revealing different peaks in the NMR ppm regions, as indicated in Figure 6a–c.

Distinct metabolites such as galactonate and galactarate were also detected under poultry manure application rate T_2_ and compared to HMDB (see Table 9).

**Figure 7:**
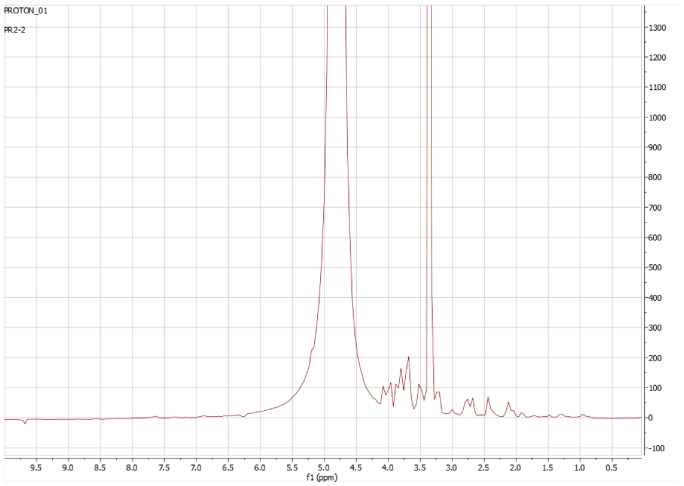

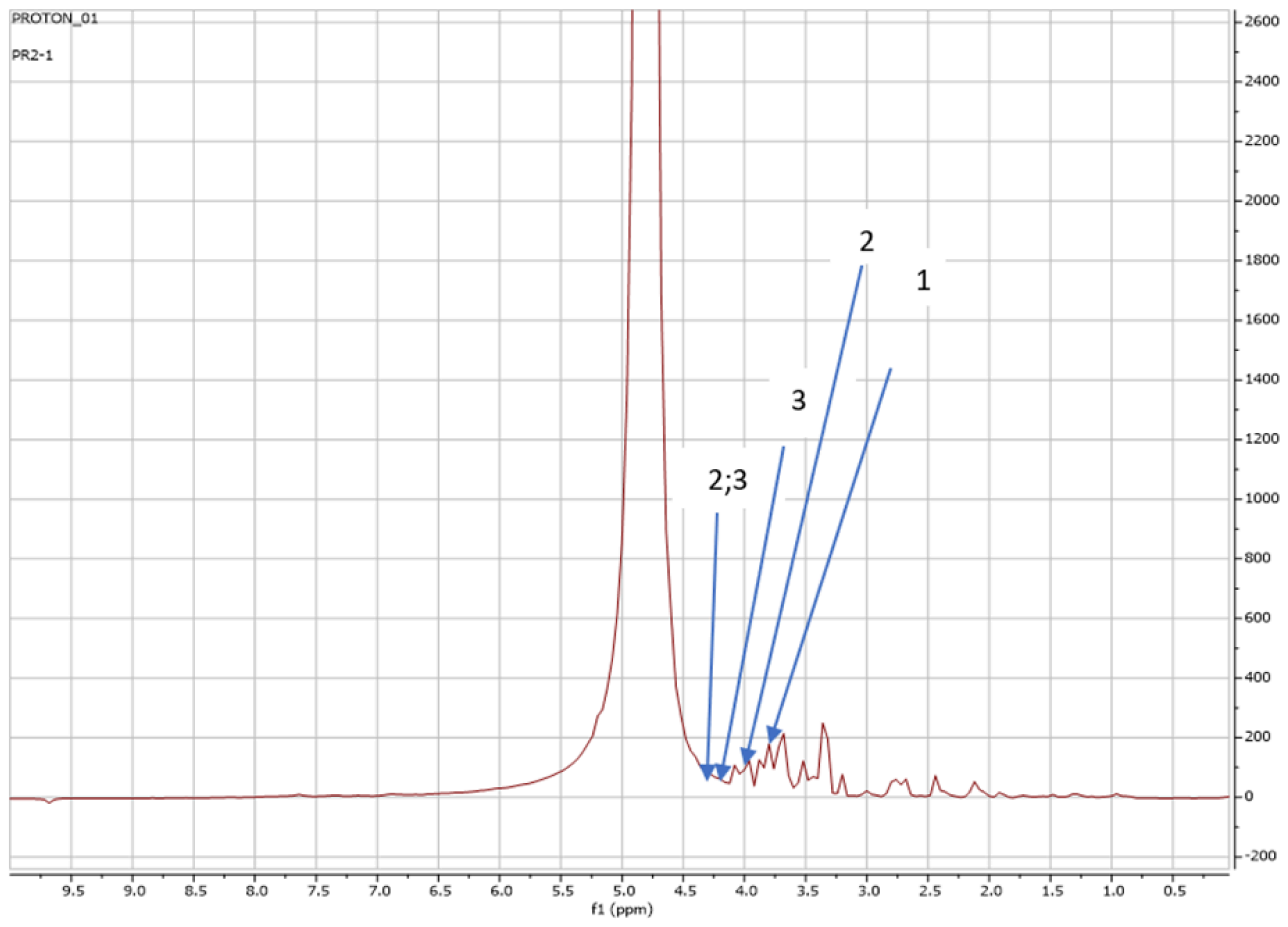
Full NMR spectra for poultry manure application rate T_2_. NMR spectra showing distinct peaks detected in fresh tomato when T_2_ poultry manure was applied: 1. 5 methoxysalicylate (3.8 ppm) 2. Galactarate (4.3, 3.9 ppm) 3. Galactonate (4.3, 4.2 ppm).

To detect and identify the secondary metabolites of field-grown tomatoes, NMR spectra for the current study were developed using Mestrenova software, revealing different peaks in the NMR ppm regions, as indicated in Figure 7.

Distinct metabolites such as uridine, acetaminophen, UDP-glucose, glutamine, and NADP+ were also detected under poultry manure application rate T_3_ and compared to HMDB (see Table 10).

**Figure 8:**
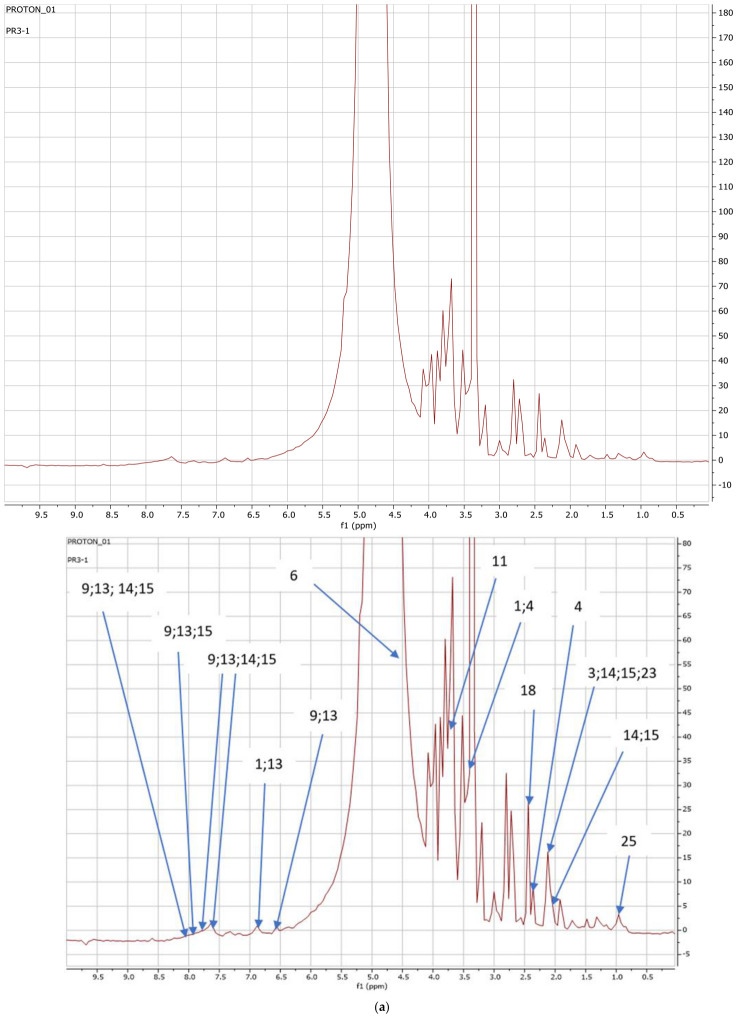

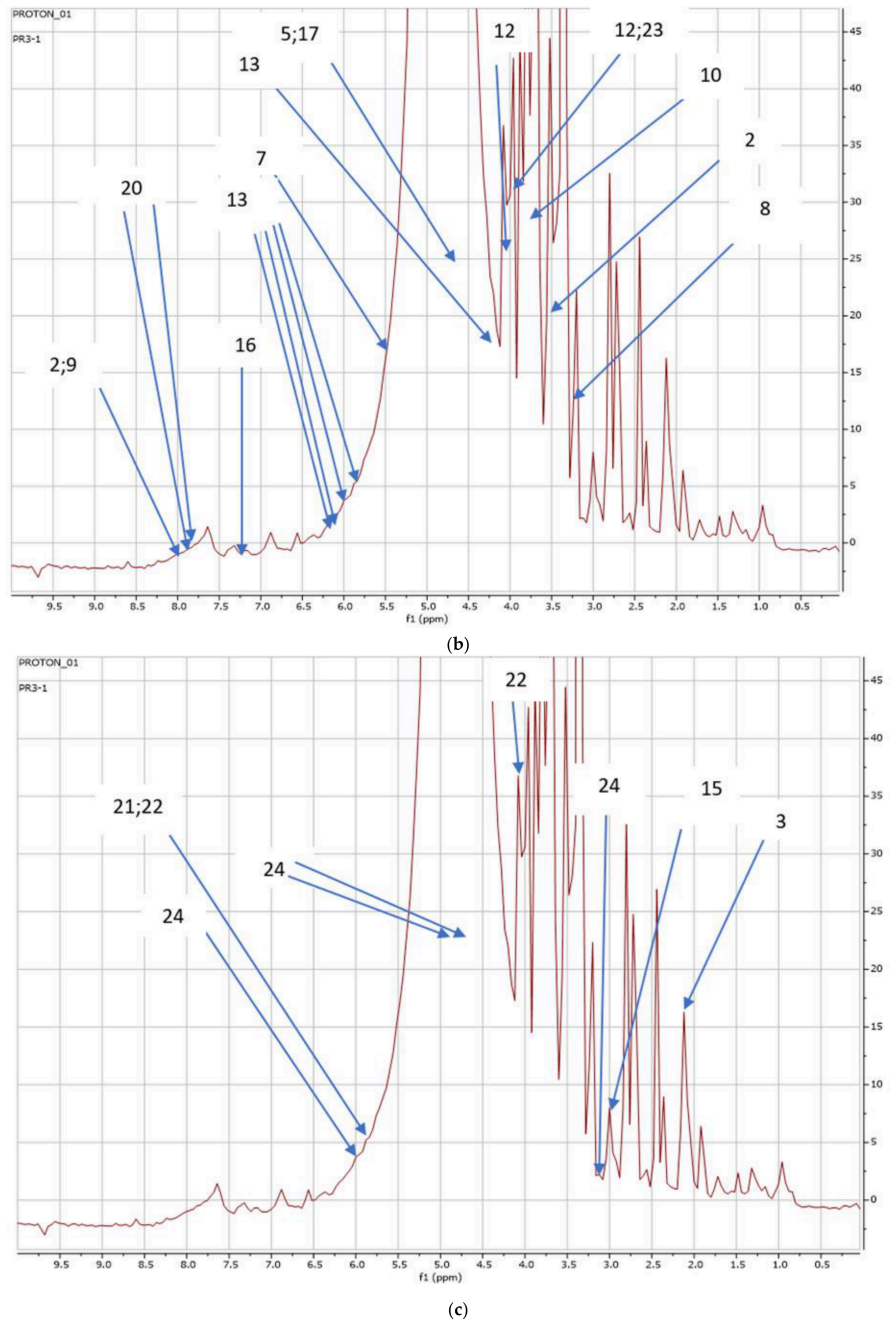
NMR full spectra for poultry manure application rate T_3_. (**a**) NMR spectra showing distinct peaks detected in fresh tomato when T_3_ poultry manure was applied: 1. 3.4-dehydroxybenzeneacetate (6.9, 3.4 ppm); 3. Acetaminophen (2.1 ppm); 4. Acetoacetate (3.4, 2.3 ppm); 6. Ascorbate (4.5 ppm); 9. Glutamine (8.3, 8.2, 8.1, 8.0, 7.9, 7.8, 7.7, 7.6, 6.6 ppm); 11. Glycine (3.6); 13. N-acetlyglutamine (8.0, 7.9, 7.8, 7.7, 7.6, 7.5, 7.4, 6.9, 6.8, 6.7, 6.6, 6.5, 6.4, 6.3, 6.2, 6.1, 6.0, 5.9, 5.7, 5.6, 4.2 ppm); 14. N-acetylglycine (8.0, 7.8, 7.7, 7.6, 2.1, 2.0 ppm); 15. N-acetlyornithine (8.0, 7.9, 7.8, 7.7, 7.6, 3.0, 2.1, 2.0 ppm); 18. N-cabormyl-B-alanine (2.4 ppm); UDP-N-acetylglusamine (2.1, 3.9); 25. Valine (1.0 ppm). (**b**) NMR spectra showing distinct peaks detected in fresh tomato when T_3_ poultry manure was applied: 2. 3-methylxanthine (8.0, 3.5 ppm); 5. ADP (4.6 ppm); 7. Chologenate (5.3 ppm); 8. Choline (3.2 ppm); 9. Glutamine (8.3, 8.2, 8.1, 8.0, 7.9, 7.8, 7.7, 7.6, 6.6 ppm); 10. Glutaric acid monomethyl ester (3.7 ppm); 12. Glycylproline (4.0, 3.9 ppm); 13. N-acetlyglutamine (8.0, 7.9, 7.8, 7.7, 7.6, 7.5, 7.4, 6.9, 6.8, 6.7, 6.6, 6.5, 6.4, 6.3, 6.2, 6.1, 6.0, 5.9, 5.7, 5.6, 4.2 ppm); 16. N-acetlyornithine (8.0, 7.9, 7.8, 7.7, 7.6, 3.0, 2.1, 2.0 ppm); 17. N-acetlyserotonin (7.2, 1.9 ppm); 20. Tartrate (4.3 ppm); 23. UDP-glucoronate (5.6, 4.1 ppm). (**c**) NMR spectra showing distinct peaks detected in fresh tomato when T3 poultry manure was applied: 3. Acetaminophen (2.1 ppm); 15. N-acetlyornithine (8.0, 7.9, 7.8, 7.7, 7.6, 3.0, 2.1, 2.0 ppm); 21. UDP-glucose (5.6 ppm); 22. UDP-glucoronate (5.6, 4.1 ppm); 24. Uridine (5.9, 4.4, 4.3, 3.1 ppm).

To detect and identify the secondary metabolites of field-grown tomatoes, NMR spectra for the current study were developed using Mestrenova software, revealing different peaks in the NMR ppm regions, as indicated in Figure 8a–c.

In addition, 1.3-dihydroxyacetone, 3-hydroxyisovalerate, cellobiose, creatine phosphate, epichatechin, galactose, maltose, O-acetylcarnitine, O-acetylcholine, pantothenate, phenylacetate, ribose, riboflavin, succinylacetone, and theophylline were detected across different rates of poultry manure.

## 4. Discussion

### 4.1. Distinct Metabolites Detected Across Different Rates of Biochar

Inosine is a purine metabolite reported to be linked to energy metabolism and stress signals, indicating active nucleotide recycling under moderate nutrient amendment [39,40]. Additionally, IMP acts as a precursor for both adenylate and guanylate nucleotides, and its degradation products include allantoin [40]. Its salvage pathway is activated to recycle nucleotides [41]. This suggests that the detection of inosine in biochar-cultivated tomatoes could reflect either enhanced purine turnover or a specific response to altered root zone oxygen dynamics. Inosine levels increased during sulfur deficiency in *Arabidopsis* to maintain homeostasis [42]. In relation to biochar amendment, the improved nutrient retention properties may paradoxically create localized nutrient gradients, which could potentially influence such metabolic adaptations. According to Noctor et al. [43], NAD+ and its reduced form (NADH) constitute the principal redox couple in cellular metabolism, participating in over 300 enzymatic reactions. The study found that the NAD+/NADH ratio reflects the metabolic state of cells, with higher ratios typically indicating active catabolic processes. A study by Zhu et al. [44] demonstrated that NAD+ biosynthesis increased during fruit ripening in tomato, correlating with enhanced respiratory activity and ethylene production. This suggests that NAD+ detection in fresh tomatoes could be linked to development regulation, potentially modified by biochar amendment. NAD+ serves as a substrate for poly (ADP-ribose) polymerase (PARP) enzymes involved in DNA repair and stress signaling [45]. According to their findings, environmental stresses that induce DNA damage can lead to NAD+ depletion, making its maintenance crucial for stress resilience.

Malonate (malonic acid) is a specialized metabolite in the plant metabolome profile [46]. According to a study by Kai et al. [47], malonate serves dual roles as a competitive inhibitor of succinate dehydrogenase in the TCA cycle and as a precursor for malonate secondary metabolites. Its accumulation can occur under conditions where carbon flux exceeds respiratory capacity. Bovy et al. [48] revealed that tomato fruits contain substantial amounts of malonate flavonoids, which enhance both stability and bioactivity. In the current study, the detection of malonate could indicate the increased production of these conjugated compounds, potentially in response to biochar-induced changes in carbon metabolism. In plants, malonate serves as the primary extender unit for polyketide biosynthesis, and its elevated pool could support increased production of important secondary metabolites, including certain phytoalexins and pigments [49].

T_2_ (10 t/ha) profile marked a significant shift towards specialized secondary metabolism and stress-related compounds, with the distinct emergence of epicatechin and chlorogenate. According to Bovy et al. [50], epicatechin belongs to the flavanol subclass of flavonoids and serves as a direct precursor for proanthocyanidin (condensed tannin) biosynthesis in tomato fruit peel. The above study found that epicatechin accumulation is regulated and peaks during the mature green stage, contributing to both fruit pigmentation and pathogen defense. A previous study by Muir et al. [51] revealed that the expression of genes encoding anthocyanidin reductase (ANR) and leucoanthocyanidin reductase (LAR), enzymes responsible for epicatechin synthesis, increases under moderate abiotic stress. This suggests that in the current study, epicatechin detection in tomatoes treated with 10 t/ha biochar may reflect early pigment readiness for the mature tomato fruits compared to the 5 t/ha (T_1_) treatment. A recent study by Manawasinghe et al. [52] agrees with the current findings that higher biochar doses (>8 t/ha) increase soil organic carbon sufficiently to stimulate lignin-degrading microbial communities, which in turn release phenolic compounds that may act as elicitors for plant secondary metabolism. On the contrary, Qu et al. [53] reported that flavonoids like epicatechin accumulate in response to altered nutrient stoichiometry, particularly changes in the nitrogen-to-carbon ratio. Therefore, the higher biochar dose (10 t/ha) likely provides greater cation exchange capacity and nutrient retention, possibly creating a more pronounced nutrient sink effect that redirects carbon toward secondary metabolite production.

Chlorogenate (chlorogenic acid) is one of the metabolites that have value in the nutraceutical market and is found in so-called superfoods [54]. Chlorogenates possess recognized human health benefits, such as antioxidant, antiviral, hepatoprotective, and antihypoglycemic properties [55], as well as anti-inflammatory [56], anticancer [57,58,59,60], antidiabetic [61], antihypertensive [62], and anti-neurodegenerative activities [63,64,65]. Bagdas [66] defined chlorogenate as a major phenolic compound in tomatoes, known for its antioxidant and anti-inflammatory properties. Another study by Lopez-Hidalgo et al. [67] reported that chlorogenic acid functions as both an antioxidant buffer and a signal molecule in plant stress response. The above study documented a positive correlation between CGA accumulation and antioxidant capacity in tomato fruits under environmental stress. The detection of chlorogenate at a biochar (10 t/ha) dose suggests that a higher application may enhance phenylpropanoid pathway flux. Furthermore, a study by Niggeweg et al. [68] stated that chlorogenic acid (CGA) is the predominant hydroxycinnamic acid conjugate in tomato, synthesized via the phenylpropanoid pathway through the esterification of caffeoyl-CoA and quinic acid. The above-mentioned study found that CGA constitutes up to 70% of the soluble phenolic pool in certain tomato cultivars and serves multiple physiological roles. Moreover, Mandal et al. [69] established that CGA accumulates in response to UV-B radiation and pathogen attack, acting as a photo-protectant and antimicrobial compound. According to their findings, biochar-induced changes in soil properties and plant vigor may modify light reflection or plant susceptibility to pathogens, ultimately stimulating CGA biosynthesis as a protective response. The 20 t/ha profile introduced another layer of complexity, featuring metabolites such as kynurenine, indole-3-acetate, glycine, etc. A study by Watanabe et al. [70] reported that the kynurenine pathway in plants serves as an alternative route for tryptophan metabolism under conditions where the primary indole-3-acetic (IAA) biosynthetic pathways are saturated or disrupted. The absence of kynurenine at lower rates (5 & 10 t/ha) suggests that this high application rate may perturb normal tryptophan metabolism, potentially through alterations in soil nitrogen dynamics or through biochar-induced changes in rhizosphere redox conditions. According to Badawy [71], kynurenine is a key intermediate in the plant tryptophan degradation pathway, specifically in the oxidative route of indole-3-acetic acid (IAA) biosynthesis and is associated with specific developmental stages and environmental stress responses, particularly those involving oxidative stress and nutrient imbalances. Furthermore, Wang et al. [72] demonstrated that kynurenine can function as a signal molecule in plant pathogen interactions and modulate defense responses through interactions with reactive oxygen species. According to Zhao [73], indole-3-acetate is the primary natural auxin in plants, regulating virtually every aspect of plant growth and development, including cell elongation, division, and differentiation. The abovementioned study emphasized that free IAA levels are tightly controlled through biosynthesis, conjugation, degradation, and transport, with perturbations often indicating significant physiological adjustments. Sun et al. [74] indicated that soil amendments can significantly influence root architecture and IAA homeostasis through direct effects on plant physiology and indirect effects via soil microbiome alterations. The presence of distinct IAA at 20 t/ha biochar suggests that the application rate may have exceeded a threshold for normal auxin regulation, potentially due to changes in soil microbial communities that might have produced or metabolized IAA [75]. Furthermore, Gravel et al. [76] demonstrated that high biochar application rates (>15 t/ha) can adsorb and subsequently release plant growth regulators, including auxin-like compounds. Cao et al. [77] demonstrated that winter forage crops, particularly Chinese milk vetch, significantly increased soil nitrogen content and AMF (*Arbuscular mycorrhizal* Fungi) spore density in paddy fields, with claroideoglomus emerging as the dominant AMF genus. The detection of free IAA at 20 t/ha could result from either enhanced plant biosynthesis as a response to an altered root environment or from biochar-mediated effects in the rhizosphere. According to a study by Lancien et al. [78], glycine serves as a central metabolite in photorespiration, glutathione synthesis, and one-carbon metabolism, and its accumulation often reflects adjustments in photorespiratory flux, particularly under conditions that affect the balance between photosynthesis and respiration. Busch et al. [79] found that glycine levels increase in response to nitrogen limitations and oxidative stress, serving as both a compatible osmolyte and a precursor for antioxidant synthesis. The distinct presence of glycine at a high application rate (20 t/ha) biochar may indicate that while increasing total nitrogen availability, it might create temporary nutrient imbalances or induce mild stress that stimulates photorespiration and amino acid redistribution.

### 4.2. Common Metabolites Detected Across Different Rates of Biochar

A recent study by Nemadodzi and Managa [19] was the first to detect allantoin (a secondary metabolite) in the leaves of *Amaranthus*, which to date has not been researched in detail. It has been assumed that the synthesis and release of allantoin make it the main protective metabolite used by *Amaranthus* species grown in open fields and greenhouses to defend against abiotic and biotic stresses. Their findings are in agreement with a study by Takagi et al. [80], which found that allantoin functions as a key metabolite in the purine catabolism pathway and accumulates under abiotic stress conditions such as drought, salinity, and nutrient deficiency. The study found that allantoin serves as an efficient nitrogen transport compound and contributes to scavenging reactive oxygen species, thereby mitigating oxidative damage. A recent study by Watanabe et al. [81] revealed that allantoin enhances salinity tolerance in *Arabidopsis* by modulating the expression of genes involved in abscisic acid signaling and antioxidant biosynthesis. This suggests that the detection of allantoin in biochar-amended tomatoes could indicate an adaptive response to improved but altered rhizosphere conditions. Furthermore, Yasmeen et al. [82] demonstrated that allantoin accumulation is linked to sulfur metabolism and glutathione homeostasis, reinforcing its role in redox balance. Moreover, a recent study by Joseph et al. [83] reported that biochar application can modify soil microbial communities and enhance the nitrogen cycle, potentially influencing plant nitrogen metabolism. This may explain the observed high concentration of allantoin accumulation in tomatoes, as biochar could promote nitrogen assimilation and purine turnover. Glutamine and asparagine are key amino acids in nitrogen assimilation, reflecting improved nitrogen uptake and utilization [84]. According to a study by Masclaux-Daubresse et al. [85], glutamine and asparagine serve as the primary organic nitrogen transport and storage compounds in most plants, dominating the nitrogen composition of phloem and xylem sap, and facilitating efficient nitrogen mobilization from source to sink tissues. Gaufichon et al. [86] revealed that glutamine synthesis, catalyzed by glutamine synthetase (GS), is the central entry point for inorganic nitrogen into organic metabolism. Furthermore, transgenic tomatoes with enhanced GS activity exhibited improved nitrogen use efficiency and fruit yield [86]. This suggests that increased glutamine levels in biochar-amended tomatoes may indicate enhanced nitrogen assimilation. The high levels of sugars and sugar derivatives such as sucrose, maltose, cellobiose, arabinose, fucose, and ribose found in the current study indicate significant alterations in carbon partitioning and carbohydrate metabolism. According to a study by Ibrahim et al. [87], biochar-amended soils often lead to increased soluble sugar content in plants, which can be linked to improved water retention and nutrient availability, reduced carbon expenditure on stress mitigation, and greater allocation to storage and fruit development. A study by Motseo and Nemadodzi [88] coincided with that reported by Gamba et al. [89], whereby it was discovered that the application of organic manure induces the presence of sucrose. 1,3-dimethylurate is a methylated derivative of uric acid and is primarily known as an intermediate in the purine degradation pathway in plants and mammals [90]. Its presence in tomato fruit suggests active purine metabolism and potential oxidative stress response mechanisms. The accumulation of methylated uric acid derivatives has been linked to enhanced nitrogen use efficiency and protection against reactive oxygen species under abiotic stress conditions [90]. Biochar-amended soils are known to improve nutrient availability, particularly nitrogen, which may stimulate purine turnover and subsequent methylation reactions, leading to the observed accumulation of 1,3-dimethylurate [91]. The consistent presence of 1,3-dimethylurate across biochar rates may indicate a biochar-induced moderation of oxidative stress in tomato plants. Similar findings were reported by Akhtar et al. [92], who observed increased levels of methylated purine metabolites in *Solanum lycopersicum* under biochar-amended drought conditions. In a study on tomato metabolomics under biochar amendment, Xizong et al. [93] also identified dimethylurate derivatives in leaf tissue, correlating them with improved photosynthetic efficiency and reduced hydrogen peroxide accumulation. Similarly, Ali et al. [94] reported elevated purine metabolites in rice grains grown in biochar-amended soils, associating them with enhanced nitrogen assimilation and stress tolerance. These studies support that methylated purines, including 1,3-dimethylurate, are part of a conserved metabolic adaptation to improved soil conditions and stress mitigation. Furthermore, a study by Motseo and Nemadodzi [88] showed that 1,3-Dimethylurate is a naturally occurring compound found in tea, coffee, and a few medications and has been reported to have numerous health benefits. For instance, 1,3-Dimethylurate possesses antioxidant and anti-inflammatory properties and potentially reduces the risk of chronic diseases such as cardiovascular disease, cancer, and neurodegenerative disorders [95]. Additionally, 1,3-Dimethylurate may improve respiratory function, enhance cognitive performance, and support weight management [88].

### 4.3. Distinct Metabolites at Specific Poultry Manure Rates

Histamine and isoeugenol were detected at 10 t/ha. Roshchina [96] reported that histamine is involved in plant defense signaling, while isoeugenol is a volatile phenylpropene with antimicrobial properties, suggesting the activation of defense pathways. A study by Pramai et al. [97] revealed that histamine in plant tissues originates from endogenous biosynthesis via histidine decarboxylase activity and microbial production in the rhizosphere, followed by plant uptake. Its distinct detection at 10 t/ha at a lower concentration suggests a specific threshold effect where moderate manure application creates conditions favorable for histamine accumulation. A study by Ishihara et al. [98] demonstrated that isoeugenol biosynthesis is strongly regulated in response to biotic stress, particularly fungal pathogen attack, where it exhibits significant antifungal properties. At 20 t/ha, 5-methoxysalicylate and galactarate were found, both linked to stress response and cell wall metabolism [99]. According to Vlot et al. [100], the presence of 5-methoxysalicylate in tomato fruits at 20 t/ha may indicate systemic defense activation originating from root zone interactions with the manure-amended soil, representing a different defensive strategy than the isoeugenol-based response observed at 10 t/ha. Studies by Anamika et al. [101] and Sharma et al. [102] revealed that 5-methoxysalicylate accumulation often occurs under conditions of sustained biotic stress or in response to specific microbial elicitors in the rhizosphere. Avci et al. [103] reported that galactarate (mucic acid), the dicarboxylic acid oxidation product of galactose, accumulates under conditions of enhanced carbohydrate oxidation or as a response to oxidative stress. Keller and Pharr [104] reported that galactarate accumulates in plant tissue during carbon excess conditions or when carbohydrate metabolism is perturbed by environmental factors. Its distinct presence at 20 t/ha PM suggests that this application rate may create conditions of carbohydrate surplus or metabolic imbalance that differ from those at 10 or 30 t/ha. ADP, glutamine, valine, and UDP-glucose were prominent at 30 t/ha, indicating active energy and carbohydrate metabolism. UDP-glucose serves as the central nucleotide sugar in plant metabolism, functioning as the activated glucosyl donor for sucrose, cell wall polysaccharides, glycoproteins, and numerous specialized metabolites [105]. According to Ruan [106], UDP glucose levels increased during periods of enhanced sink strength and active biosynthesis, particularly in developing fruits, where it supplies precursors for structural and storage carbohydrates. The presence of UDP-glucose in tomato fruits at 30 t/ha may indicate not only abundant carbon resources but also metabolic regulation that optimizes resource allocation under conditions of substantial organic amendment [107]. Valine belongs to the branched-chain amino acid (BCAA) group and plays crucial roles in protein synthesis, stress responses, and as a precursor for secondary metabolites [108]. Its accumulation typically reflects active protein synthesis or specific stress adaptation responses, particularly under conditions of nitrogen abundance. According to the findings of the current study, its detection specifically at 30 t/ha may represent an integrated strategy for utilizing abundant nitrogen resources for both growth and defense purposes [109].

### 4.4. Common Metabolites Across Poultry Rates

Riboflavin (vitamin B2) and pantothenate (vitamin B5) are essential cofactors in energy metabolism, reflecting enhanced metabolic activity. According to a study by Kubicek et al. [110], cellobiose is a disaccharide composed of two β-(1-4)-linked glucose units and serves as a major intermediate in cellulose degradation. In plant tissues, its accumulation is uncommon and typically indicates either specific developmental stages or responses to external biotic factors. Chen et al. [111] reported that soil amendments rich in organic matter, such as poultry manure, profoundly stimulate cellulolytic microbial communities in the rhizosphere, which ultimately convert cellulose into cellobiose, which is subsequently taken up by plant roots. The constant detection of cellobiose across all poultry manure rates suggests a direct microbial-plant interaction pathway unique to organic fertilization. Furthermore, Jones et al. [112] established that cellobiose can act as a signal molecule in plant-microbe interactions, potentially priming plant defense responses. The presence of cellobiose in tomato fruits in the current study may reflect systemic effects originating from enhanced rhizosphere interactions between plant roots and manure-stimulated microbes. Riboflavin is an essential water-soluble vitamin that functions as a precursor for the coenzymes flavin mononucleotide (FMN) and flavin adenine dinucleotide (FAD), which are crucial for electron transfer in numerous metabolic reactions [113]. According to Shumo et al. [114], poultry manure application significantly increases riboflavin content in crops through two primary mechanisms: enhanced plant biosynthesis stimulated by improved nutrient status and direct contribution from manure-derived microbes that synthesize riboflavin. The consistent presence of riboflavin indicates a reliable nutritional enhancement effect [115]. A study by LeBlanc [116] revealed that riboflavin in plants serves not only as a vitamin but also as a photo-protectant and signal molecule involved in stress responses. The poultry manure-induced riboflavin accumulation may represent both a nutritional improvement and an adaptive response to the richer and more variable microbial environment. Pantothenate is the precursor for coenzyme A (CoA) and acyl carrier protein (ACP), making it essential for fatty acid metabolism, citric acid cycle operation, and numerous acetylation reactions. In plants, pantothenate biosynthesis is tightly regulated and responds to metabolic demand [117]. The consistent detection across poultry manure treatments suggests that it creates high metabolic activity in tomatoes, which aligns with findings by Eichler et al. [118] that organic manure increases the activity of enzymes involved in energy transduction and carbon metabolism. A study by Shi et al. [119] stated that integrating biotechnology with traditional agricultural practices offers a critical route for future innovation, particularly in response to climate change and population growth.

## 5. Conclusions

This study demonstrated that both organic amendments (biochar and poultry manure) significantly influenced the metabolomic profile of tomato fruits and induced greater metabolic diversity in tomato fruit. The application of biochar at increasing rates (5, 10, 20 t/ha) resulted in a dose-dependent expansion of the tomato metabolome profile, with 84, 100, and 110 metabolites detected, respectively. Similarly, poultry manure applications at 10, 20, and 30 t/ha induced substantial metabolic alterations, with the highest metabolite count (103) recorded at 30 t/ha. The detection of health-promoting metabolites across organic treatments, such as allantoin (an antioxidant for stress protection), epicatechin (a flavonoid with antioxidant properties), chlorogenate (an antidiabetic), betaine (which is cardiovascular and neuroprotective), and riboflavin (an essential vitamin), suggests that organic amendments, particularly at higher application rates, may enhance the functional quality and nutraceutical value of tomato fruits. Biochar rates of 10–20 t/ha and poultry manure at 30 t/ha demonstrated the greatest metabolic diversity and production of health-beneficial compounds.

## Figures and Tables

**Table 1:** Chemical composition of pinewood biochar used in the experiments [33].

Characteristics	Biochar
pH (H_2_O)	9.23
EC (mS m^−1^)	40
Ash (mg kg^−1^)	28.40
C in ash (mg kg^−1^)	4.5
Moisture (%)	6.66
Total solids (%)	93.3
Organic matter (g kg^−1^)	905
Organic carbon (g kg^−1^)	547
Total C (g kg^−1^)	549
Total N (g kg^−1^)	0.70
C:N Ratio	776
Available P (mg kg^−1^)	4.89
Exchangeable cations cmol_(+)_ kg^−1^	
K	3.38
Na	0.53
Mg	0.89
Ca	5.23
CEC (cmol_(+)_ kg^−1^)	1.94

**Table 2:** Chemical composition of poultry manure used in the experiment [33].

Characteristics	Poultry Manure
pH (H_2_O)	7.02
Total C (%)	31.9
Total N (%)	1.6
CEC (cmol_(+)_ kg^−1^)	22.14
P (g kg^−1^)	9.68
K (g kg^−1^)	11.21
Na (g kg^−1^)	2.18
Ca (g kg^−1^)	90.6
Mg (g kg^−1^)	6.58
C:N Ratio	19.8

**Table 3:** Goodness-of-fit and predictability values of the OPLS-DA model on different rates of Biochar.

R^2^X	R^2^Y	Q^2^
0.379	0.5	0.184

**Table 4:** Distinct metabolites released per 5 (T_1_) t/ha of biochar.

Metabolite	5 (T_1_) t/ha NMR Peaks (ppm)	Concentration (Mm)	Chenomx Peak	HMDB
1. 3-hydroxy-3 methylglutarate	1.3	0.0381	2.5, 2.4, 1.3	N/A
2. 3-hydroxylsovalerate	2.4, 2.3, 1.3	7.3862	2.4, 1.3	2.35, 1.26
3. Choline	3.2	0.0562	4.1, 3.5, 3.2	3.20, 3.50, 4.10
4. Glycylproline	4.0, 3.9	0.7199	4.0, 4.3, 3.9, 3.6, 3.5, 2.3, 2.2, 2.1, 2.0, 1.9	3.95, 3.93, 3.81, 3.42, 3.40, 3.38, 3.30, 3.28, 3.26, 2.22, 2.21, 2.20, 2.19, 2.18, 1.78, 1.77, 1.76, 1.75, 1.74, 1.73, 1.72
5. Homocitrulline	6.0, 5.8, 5.7, 5.4	2.8624	6.0, 5.8, 5.7, 5.4, 3.7, 3.1, 1.9, 1.5, 1.4	N/A
6. Inosine	8.2, 8.3, 6.1	0.0587	8.3, 8.2, 6.1, 4.8, 4.4, 4.2, 3.9, 3.8	8.49, 6.10, 6.08, 4.80, 4.79, 4.78, 4.75, 4.74, 4.39, 4.38, 4.37, 4.35, 4.34, 4.33, 3.96, 3.94, 3.93, 3.92, 3.91, 3.89, 3.86, 3.84, 3.83
7. Malonate	3.2	0.3275	3.1	3.1
8. Nicotinate	7.6	0.0340	8.9, 8.2, 7.6	N/A
9. Nicotinic acid adenine dinucleotide	4.5, 4.4, 4.3	3.1407	9.1, 9.0, 8.8, 8.4, 8.1, 6.0, 4.8, 4.5, 4.4, 4.3, 4.2	3.47, 3.48, 3.49, 3.50, 3.84, 3.85, 4.10, 4.11, 4.12, 4.13, 4.17, 4.18, 4.19, 4.20, 4.21, 4.23, 4.24, 4.25, 4.26, 4.27, 4.31, 4.35, 4.36, 4.37, 4.38, 4.64, 4.65, 6.09, 6.10, 8.30, 8.31, 8.48, 8.98, 8.99, 9.33, 9.34, 9.57, 9.58
10. N-Methylhydantoin	4.1, 2.9	0.2154	4.1, 2.9	4.08, 2.92
11. Nα-acetyllysine	7.5, 2.1, 2.0	0.0649	8.0, 7.5, 3.0, 4.1, 2.0, 1.8, 1.7, 1.4	4.16, 4.15, 4.14, 3.80, 3.79, 3.78, 3.77, 3.76, 3.01, 3.00, 2.99, 2.98, 2.97, 2.55, 2.54, 2.53, 2.53, 2.52, 2.51, 2.50, 2.49, 2.02, 1.89, 1.82, 1.81, 1.80, 1.79, 1.78, 1.77, 1.70, 1.69, 1.68, 1.67, 1.66, 1.43, 1.42, 1.41, 1.40, 1.39
12. O-acetylcholine	3.2, 2.1	0.0763	4.6, 3.2, 3.1	N/A
13. Trans-Aconitate	3.4	0.1064	6.6, 3.4	6.41, 6.38, 6.35, 3.47, 3.44, 3.41
14. UDP-Glucuronate	5.6, 4.1	0.7760	7.9, 6.0, 5.6, 4.4, 4.3, 4.2, 4.1, 3.8	N/A

**Table 5:** Distinct metabolites released at a biochar application rate of 10 t/ha (T_2_).

Metabolite	10 t/ha NMR Peaks (ppm)	Concentration (Mm)	Chenomx Peaks	HMDB
1. 1-Methylnicotinamide	4.5	0.5739	9.3, 9.0, 8.9, 8.2, 4.5	N/A
2. 2′-deoxyguanosine	6.3	0.0002	8.0, 6.3, 4.6, 4.1, 3.8, 2.8, 2.5	8.31, 6.31, 6.30, 6.29, 6.28, 6.27, 4.67, 4.66, 4.65, 4.64, 4.63, 4.13, 4.11, 3.82, 3.81, 3.80, 3.79, 3.76, 3.75, 3.74, 3.73, 2.84, 2.83, 2.82, 2.81, 2.80, 2.59, 2.57, 2.55, 2.53
3. 2-deoxyinosine	4.6	0.6905	8.3, 8.2, 6.5, 4.6, 4.2, 3.8, 2.8, 2.6	8.19, 7.99, 6.46, 6.45, 6.44, 6.43, 6.42, 6.31, 6.30, 6.29, 6.28, 6.27, 3.88, 3.87, 3.86, 3.84, 3.83, 3.82, 3.75, 2.27, 2.26, 2.25, 2.24, 2.23, 2.22
4. 3-hydroxyphenylacetate	6.8	0.0017	7.2, 6.8, 3.5	N/A
5. Acetamide	7.7, 6.9, 6.5, 6.4	0.0052	7.7, 6.8, 2.0	1.99
6. AMP	4.5	0.1601	8.6, 8.2, 6.1, 4.8, 4.5, 4.3, 4.1, 4.0	3.49, 3.99, 4.00, 4.01, 4.02, 4.03, 4.04, 4.05, 4.61, 4.62, 4.63, 4.78, 4.79, 4.80, 6.08, 6.10, 8.18, 8.49
7. Anserine	7.2, 7.0, 7.1, 7.3, 7.4, 7.6, 7.5, 7.7, 7.9, 7.8, 3.8	0.0027	8.3, 8.2, 7.1, 4.5, 3.8, 3.2, 3.0, 2.7, 2.6	N/A
8. Carnosine	4.5	0.4869	8.1, 7.1, 4.5, 3.2, 3.0, 2.7, 2.6	N/A
9. Chlorogenate	7.2, 6.9, 6.4	0.0344	7.6, 7.0, 7.1, 6.9, 6.1, 5.3, 4.2, 3.9, 2.2, 2.0	N/A
10. Citrulline	7.8, 7.5, 7.6, 7.3, 7.2, 7.0, 7.1, 6.8, 6.9, 6.7, 6.6, 6.5, 6.4, 6.3, 6.2, 6.1	0.0344	6.5, 3.7, 3.1, 1.9, 1.8, 1.6, 1.5	3.86, 3.85, 3.84, 3.80, 3.79, 3.78, 3.77, 3.76, 3.15, 3.14, 3.13, 3.12, 3.11, 2.65, 2.64, 2.63, 2.62, 2.61, 2.60, 2.59, 1.91, 1.90, 1.89, 1.88, 1.87, 1.86, 1.85, 1.55, 1.54, 1.53, 1.52, 1.51
11. Cytidine	4.2	0.0242	7.8, 6.1, 5.9, 4.3, 4.2, 4.1, 3.9, 3.8	N/A
12. Epicatechin	5.0, 4.4	0.0573	7.1, 7.0, 6.9, 6.1, 5.0, 4.3, 2.9, 2.8	N/A
13. Ethylene glycol	3.7	0.0300	3.7	N/A
14. Ferulate	6.9, 6.4, 3.9	0.0117	7.3, 7.1, 6.9, 6.4, 3.9	N/A
15. Galactose	5.3, 4.6	0.3701	5.3, 4.6, 4.1, 4.0, 3.9, 3.8, 3.7, 3.6, 3.5	3.51, 3.52, 3.53, 3.65, 3.66, 3.74, 3.74, 3.80, 3.81, 3.82, 3.83, 3.84, 3.94, 3.95, 3.96, 4.03, 4.04, 4.11, 4.12, 4.13, 4.19, 4.20, 5.20, 5.21, 5.22, 61.79, 70.68, 72.81, 73.72, 76.26, 93.99
16. Glycolate	3.9	0.0123	3.9	N/A
17. N-acetylcysteine	4.4	0.1871	8.0, 4.4, 2.9, 2.1	3.86, 3.85, 3.84, 3.80, 3.79, 3.78, 3.77, 3.76, 3.15, 3.14, 3.13, 3.12, 3.11, 2.65, 2.64, 2.63, 2.62, 2.61, 2.60, 2.59, 1.91, 1.90, 1.89, 1.88, 1.87, 1.86, 1.85, 1.55, 1.54, 1.53, 1.52, 1.51
18. N-Acetylglutamate	4.1	0.0292	8.1, 4.1, 2.2, 2.0, 1.9	4.11, 4.10, 4.09, 3.04, 3.03, 3.02, 3.01, 3.00, 2.99, 2.98, 2.23, 2.22, 2.21, 2.02, 1.89, 1.88, 1.87, 1.86, 1.85, 1.84, 1.83
19. N-acetylserotonin	7.2, 7.1, 6.8	0.0005	9.9, 7.9, 7.4, 7.2, 7.1, 6.8, 3.5, 2.9, 1.9	N/A
20. N-acetyltyrosine	7.7, 7.6, 1.9	0.0025	7.7, 7.1, 6.8, 4.4, 3.1, 2.8, 1.9	0.01, 0.02, 0.03, 0.04, 0.05, 0.07
21. NAD+	6.1, 6.0, 4,5, 4.4	0.2427	9.3, 9.1, 8.8, 8.4, 8.2, 6.1, 6.0, 4.8, 4.4, 4.3, 4.2	2.84, 2.85, 2.86, 2.87, 3.47, 3.48, 3.49, 3.50, 3.83, 3.84, 3.85, 3.86, 4.18, 4.19, 4.20, 4.21, 4.35, 4.36, 4.37, 4.38, 4.60, 4.61, 4.62, 4.63, 4.73, 4.74, 4.75, 4.76, 6.11, 6.12, 6.18, 6.19, 7.58, 7.59, 7.60, 7.61, 7.80, 7.81, 7.82, 8.14, 8.48, 9.33
22. NADH	7.0, 6.9, 6.1, 4.5, 4.4, 4.3, 4.2, 4.1, 2.8, 2.7	0.0882	8.5, 8.2, 7.0, 6.1, 6.0, 4.8, 4.7, 4.5, 4.4, 4.3, 4.2, 4.1, 2.8, 2.7	N/A
23. NADP+	4.5, 4.4, 4.3, 4.2	0.0864	9.3, 91, 8.8, 8.4, 8.2, 8.1, 6.1, 6.0, 5.0, 4.6, 4.5, 4.4, 4.3, 4.2	3.75, 3.76, 3.77, 3.78, 3.88, 3.89, 3.90, 3.91, 3.92, 4.01, 4.02, 4.03, 4.04, 4.05, 4.23, 4.24, 4.25, 4.26, 4.31, 4.32, 4.33, 4.34, 4.35, 4.50, 4.51, 4.52, 4.53, 4.54, 5.95, 5.96, 5.97, 5.98, 6.23, 6.24, 6.25, 8.09, 8.10, 8.16, 8.17, 8.18, 8.22, 8.29, 8.98, 9.00, 9.01, 9.03, 9.04, 9.05, 9.46, 9.47
24. Nicotinomide-N-oxide	7.6	30.3552	8.7, 8.5, 8.4, 8.1, 7.7, 7.6	7.43, 7.44, 7.45, 8.20, 8.21, 8.22, 8.82, 8.91
25. N-phenylacetylphenylalanine	3.6, 4.5	0.3426	7.7, 7.3, 7.2, 7.1, 4.5, 3.6, 3.5, 3.2, 2.9	N/A
26. p-Cresol	2.3	0.0093	7.1, 6.8, 2.3	N/A
27. pyroglutamate	4.2	0.0547	7.7, 4.2, 2.4, 2.0	N/A
28. UDP-galactose	6.0, 5.6, 4.3, 4.2	0.0574	7.9, 5.6, 4.3, 4.2, 4.1, 3.9, 3.8, 3.7	N/A
29. Valine	1.0	0.0007	3.6, 2.3, 1.0	N/A

**Table 6:** Distinct metabolites released at a biochar application rate of 20 t/ha (T_3_).

Metabolite	20 t/ha NMR Peaks (ppm)	Concentration (Mm)	Chenomx Peaks	HMDB
1. ADP	4.4	2.2820	8.5, 8.3, 6.1, 4.7, 4.6, 4.4, 4.3, 4.2	N/A
2. Creatinine	4.1	0.2107	4.1, 3.0	4.11, 4.07, 4.06, 4.02, 3.03
3. Galactarate	4.3, 3.9	0.0501	4.3, 4.0	N/A
4. Galactonate	4.3	0.1720	4.3, 4.0, 3.7, 3.6	N/A
5. Glucarate	4.1	0.1036	4.2, 4.1, 3.9	N/A
6. Glycine	3.6	0.1525	3.6	N/A
7. Glycocholate	3.8, 0.9, 0.7	1.6315	7.9, 4.0, 3.9, 3.8, 3.7, 3.5, 2.5, 2.2, 2.0, 1.9, 1.8, 1.7, 1.6, 1.4, 1.3, 1.1, 1.0, 0.9, 0.7	N/A
8. Homogentisate	3.5	0.1631	6.8, 6.7, 3.5	N/A
9. Indole-3-acetate	3.7, 3.6	0.0000	10.0, 7.6, 7.5, 7.2, 3.6	2.56, 2.57, 2.59, 2.60, 2.90, 2.91, 2.93, 2.94, 4.34, 4.35, 4.36, 7.27, 7.28, 7.29, 7.35, 7.36, 7.37, 7.38, 7.44, 7.46, 7.52, 7.53, 7.54, 7.60, 7.61, 7.62, 7.63, 7.70
10. Isoeugenol	3.9	0.1317	7.1, 6.9, 6,4, 6.2, 3.9, 1.8	1.80, 1.82, 3.86, 5.99, 6.00, 6.01, 6.02, 6.03, 6.04, 6.05, 6.26, 6.28, 6.79, 6.81, 6.99, 7.00, 7.01, 7.15
11. Kynurenine	3.7	0.2033	7.8, 7.4, 6.9, 6.8, 4.1, 3.7	N/A
12. N-N dimethylformamide	3.0	0.0390	7.9, 3.0, 2.9	N/A
13. N-nitrosodimethylamine	3.8	0.2446	3.8, 3.2	3.01
14. S-Adenosylhomocysteine	4.4	0.7412	8.4, 8.3, 6.1, 4.9, 4.4, 3.8, 3.1, 3.0, 2.7, 2.1	8.49, 6.06, 6.08, 4.39, 4.38, 4.37, 4.36, 4.35, 3.50, 3.49, 3.48, 3.86, 3.85, 3.84, 3.09, 3.08, 3.07, 3.06, 3.05, 2.74, 2.73, 2.72, 2.27, 2.26, 2.25, 2.24, 2.23, 2.22, 2.21, 2.20, 2.19, 2.18, 2.17, 2.16, 2.14, 2.13, 2.11, 2.10, 2.09, 2.08
15. Sarcosine	3.6, 2.7	0.0301	3.6, 2.7	N/A
16. Threonine	1.3	0.0144	4.2, 3.6, 1.3	N/A

**Table 7:** Goodness-of-fit and predictability values of the OPLS-DA model.

R^2^X	R^2^Y	Q^2^
0.842	0.183	−0.255

**Table 8:** Distinct metabolites released per 10 (T_1_) t/ha of poultry manure.

Metabolite	10t/ha NMR Peaks (ppm)	Concentration	Chenomx Peaks	HMDB
1. 2-Methylglutarate	1.0	0.0069	2.2, 2.1, 1.7, 1.0	2.54, 2.53, 2.52, 2.51, 2.50, 2.49, 2.48, 2.16, 2.15, 2.14, 1.78, 1.77, 1.76, 1.75, 1.74, 1.73, 1.72, 1.62, 1.61, 1.60, 1.59, 1.58, 1.57, 1.56, 1.08, 1.06
2. 2-phenylpropionate	1.4	0.0561	7.4, 7.3, 3.6, 1.4	7.43, 7.42, 7.41, 7.38, 7.37, 7.36, 7.35, 7.31, 7.30, 7.29, 7.28, 7.27, 3.40, 3.39, 3.37, 3.36, 1.14, 1.12
3. 3-hydroxy-3 methylglutarate	3.8, 0.9	0.0172	2.5, 2.4, 1.3	N/A
4. Dimethylamine	2.7	0.0044	2.7	2.50
5. Dimethyl sulfone	3.1, 3.2	0.0003	3.1	3.1
6. Ethelynglycol	3.7	0.0979	3.7	N/A
7. Histamine	3.0	0.3010	7.90, 7.2, 3.3, 3.0	N/A
8. Isoeugenol	3.9, 1.8	0.2533	7,1, 6.9, 6.4, 6.2, 3.9, 1.8	7.15, 6.99, 7.00, 7.01, 6.79, 6.81, 6.26, 6.28, 5.99, 6.00, 6.01, 6.02, 6.03, 6.04, 6.05, 3.86, 1.80, 1.82
9. Isoleucine	1.0	0.0250	3.7, 1.9, 1.5, 1.2, 1.0, 0.9	3.66, 2.00, 1.99, 1.98, 1.97, 1.96, 1.95, 1.94, 1.27, 1.26, 1.25, 1.24, 1.23, 1.01, 0.99, 0.94, 0.93, 0.92
10. Malonate	3.2	0.0069	3.1	3.1
11. Methionine	2.1	0.0279	3.8, 2.7, 2.2, 2.1	3.86, 3.85, 3.84, 2.64, 2.63, 2.62, 2.22, 2.21, 2.20, 2.19, 2.18, 2.17, 2.16, 2.14, 2.13, 2.12, 2.11, 2.10, 2.09, 2.08
12. Methylamine	2.6	0.0005	2.6	N/A
13. Methylguanidine	2.8	0.0072	7.0, 6.9, 2.8	2.66
14. N-Phenlyacetylglycine	3.7	0.7018	8.1, 7.4, 7.3, 3.7	N/A
15. Pyrimidine	9.1, 8.9	0.0270	9.2, 8.8, 7.6	9.13, 8.82, 8.81, 8.80, 7.61, 7.60, 7.59
16. Pyruvate	2.4	0.0027	2.4	2.36
17. Xanthine	7.9	0.0586	7.9	N/A
18. Xanthurenate	6.9	0.0036	7.5, 7.4, 7.1, 6.9	7.17, 7.16, 7.15, 7.07, 7.05, 6.88, 6.79, 6.78, 6.77, 6.71

**Table 9:** Distinct metabolites released per 20 (T_2_) t/ha of poultry manure.

Metabolites	20 t/ha NMR Peaks (ppm)	Concentration	Chenomx Peaks	HMDB
1. 5-Methoxysalicylate	3.8	0.0304	7.4, 7.1, 6.9, 3.8	7.32, 7.29, 7.26, 6.82, 6.81, 6.80, 6.79, 6.78, 6.77, 3.86
2. Galactarate	4.3, 3.9	0.3543	4.3, 3.9	N/A
3. Galactonate	4.3, 4.2	0.2578	4.3, 4.0, 3.7, 3.6	N/A

**Table 10:** Distinct metabolites released per 30 (T_3_)t/ha of poultry manure.

Metabolite	30 t/ha NMR Peaks (ppm)	Concentration	Chenomx Peaks	HMDB
1. 3,4-dehydroxybenzeneacetate	6.9, 3.4	0.0730	6.9, 6.8, 6.7, 3.4	N/A
2. 3-Methylxanthine	8.0, 3.5	0.2778	8.0, 3.5	7.82, 3.47
3. Acetaminophen	2.1	0.0478	7.2, 6.9, 2.1	7.30, 7.28, 7.02, 7.00, 6.99, 6.98, 6.77, 2.14
4. Acetoacetate	3.4, 2.3	0.3828	3.4, 2.3	N/A
5. ADP	4.6	6.8286	8.5, 8.3, 6.1, 4.7, 4.6, 4.4, 4.2	N/A
6. Ascorbate	4.5	1.9067	4.5, 4.0, 3.8, 3.7	N/A
7. Chologenate	5.3	1.0221	7.6, 7.2, 7.1, 6.9, 6.4, 5.3, 4.3, 3.9, 2,2, 2.1, 2.0	N/A
8. Choline	3.2	0.0808	4.1, 3.5, 3.2	N/A
9. Glutamine	8.3, 8.2, 8.1, 8.0, 7.9, 7.8, 7.7, 7.6, 6.6	5.5511	8.2, 6.9, 3.8, 2.5, 2.4, 2.1	3.77, 3.76, 3.75, 2.49, 2.48, 2.46, 2.45, 2.44, 2.43, 2.42, 2.40, 2.39, 2.16, 2.15, 2.13, 2.12, 2.11, 2.10, 2.09
10. Glutaric acid monomethyl ester	3.7	0.2455	3.7, 2.4, 2.2, 1.8	3.70, 2.54, 2.53, 2.52, 2.22, 2.21, 2.20, 2.19, 2.18, 2.17, 2.16, 2.15, 2.14, 1.89, 1.88, 1.87, 1.86, 1.85
11. Glycine	3.6	0.3172	3.6	N/A
12. Glycylproline	4.0, 3.9	1.1641	4.3, 4.0, 3.9, 3.6, 3.5, 2.3, 2.2, 2.1, 2.0, 1,9, 1.8	3.95, 3.93, 3.81, 3.42, 3.40, 3.38, 3.30, 3.28, 3.26, 2.22, 2.21, 2.20, 2.19, 2.18, 1.78, 1.77, 1.76, 1.75, 1.74, 1.73, 1.72
13. N-actelyglutamine	8.0, 7.9, 7.8, 7.7, 7.6, 7.5, 7.4, 6.9, 6.8, 6.7, 6.6, 6.5, 6.4, 6.3, 6.2, 6.1, 6.0, 5.9, 5.7, 5.6, 4.2	1.6349	7.5, 7.3, 6.9, 4.2, 2.4, 2.3, 2.1, 2.0, 1.9	4.16, 4.15, 4.14, 2.30, 2.34, 2.33, 2.32, 2.31, 2.29, 2.14, 2.13, 2.12, 2.11, 2.10, 2.09, 2.08, 2.02, 1.90, 1.94, 1.93, 1.92, 1.91, 1.89, 1.88
14. N-acetylglycine	8.0, 7.8, 7,7, 7,6, 2.1, 2.0	0.0306	7.7, 3.7, 2.0	3.75, 2.04
15. N-acetlyornithine	8.0, 7.9, 7.8, 7.7, 7.6, 3.0, 2.1, 2.0	0.0324	7.6, 4.2, 3.0, 2.0, 1.8, 1.7	3.75, 3.74, 3.73, 3.21, 3.20, 3.19, 1.97, 1.87, 1.86, 1.85, 1.84, 1.83, 1.82, 1.81, 1.62, 1.61, 1.60, 1.59, 1.58, 1.57, 1.56
16. N-acetlyserotonin	7.2, 1.9	0.0642	9.9, 7.9, 7.4, 7.2, 7.1, 6.9, 3.5, 2.9, 1.9	N/A
17. NADP+	4.6	17.0579	9.3, 9.1, 8.8, 8.4, 8.2, 8.1, 6.1, 6.0, 5.0, 4.6, 4.5, 4.4, 4.3, 4.2	9.47, 9.05, 9.04, 9.03, 9.01, 8.29, 8.22, 8.18, 8.17, 8.16, 8.10, 6.25, 6.23, 5.98, 5.97, 5.96, 4.53, 4.52, 4.51, 4.50, 4.35, 4.34, 4.33, 4.32, 4.31, 4.26, 4.25, 4.24, 4,05, 4,04, 4,03, 4.02, 4.01, 3.92, 3.91, 3.90, 3.89, 3.88, 3.78, 3.77, 3.76
18. N-cabormyl-β-alanine	2.4	0.4100	6.2, 3.3, 2.4	N/A
19. Tartrate	4.3	0.2032	4.3	4.34, 3.70
20. Tiglyglycine	7.9, 7.8	0.6310	7.9, 6.5, 3.8, 1.8	6.67, 6.66, 6.65, 6.64, 6.63, 3.88, 1.84, 1,78, 1.76, 1.34
21. UDP-glucose	5.6	0.8472	7.9, 6.0, 5.6, 4.4, 4.3, 4.2, 3.9, 3.8, 3.5	8.11, 6.71, 5.64, 5.63, 5.62, 4.39, 4.38, 4.37, 4.36, 4.35, 4.28, 4.27, 4.26, 4.25, 4.24, 4.23, 4.22, 4.21, 4.20, 4.19, 4.18, 4.17, 4.13, 4.12, 4.11, 3.72, 3.51, 3.50, 3.49, 3.48, 3.47,
22. UDP-glucuronate	5.6, 4.1	0.4483	7.9, 6.0, 5.6, 4.4, 4.1, 3.8, 3.6	N/A
23. UDP-N-acetlyglusamine	3.9, 2.1	0.9091	8.3, 7.9, 6.0, 5.5, 4.4, 4.3, 4.2, 4.0, 3.9, 2.8, 3.5, 2.1	8.98, 7.99, 5.97, 5.96, 4.98, 4.96, 4.43, 4.38, 4.37, 4.36, 4.27, 4.26, 4.25, 4.24, 4.23, 4.22, 4.21, 4.20, 4.19, 4.18, 4.13, 4.12, 4.11, 4.09, 4.08, 4.06, 4.05, 4.04, 4.02, 3.95, 3.80, 3.73, 3.72, 3.71, 3.70, 3.65, 3.50, 3.49, 3.48, 2.04
24. Uridine	5.9, 4.4, 4.3, 3.1	1.1867	7.9, 5.9, 4.4, 4.2, 4.1, 3.9, 3.8	9.58, 6.09, 6.08, 6.06, 4.63, 4.62, 4.61, 4.10, 4.09, 4.08, 3.92, 3.91, 3.90, 3.76, 3.75, 3.74, 3.50, 3.49, 3.48
25. Valine	1.0	0.0293	3.6, 2.3, 1.0	N/A

## Data Availability

The data presented in this study are available on request from the corresponding author due to ethical restrictions.
